# Impact of *α*-linolenic acid supplementation on long-chain *n*-3 fatty acid profiles in Western, flexitarian, vegetarian, and vegan diets

**DOI:** 10.3389/fnut.2025.1727308

**Published:** 2025-12-15

**Authors:** Lea Klein, Kristin Kipp, Stefan Lorkowski, Fabian Eichelmann, Christine Dawczynski

**Affiliations:** 1 Junior Research Group Nutritional Concepts, Institute of Nutritional Sciences, Friedrich Schiller University Jena, Jena, Germany; 2 Department for Pediatrics, Sophien- und Hufeland-Klinikum, Weimar, Germany; 3 Competence Cluster for Nutrition and Cardiovascular Health (nutriCARD) Halle-Jena-Leipzig, Jena, Germany; 4 Institute of Nutritional Sciences, Friedrich Schiller University Jena, Jena, Germany; 5 German Institute of Human Nutrition Potsdam-Rehbruecke (DIfE), Nuthetal, Germany; 6 German Center for Diabetes Research (DZD), Neuherberg, Germany

**Keywords:** PUFA, *n*-3 fatty acid, *n*-6 fatty acid, fatty acid profile, flaxseed oil, plant-based diet

## Abstract

**Introduction:**

Long-chain (LC) *n*-3 polyunsaturated fatty acids (PUFA) are critical nutrients in vegetarian and vegan diets due to the absence of fish and other animal products. *α*-Linolenic acid (ALA) is the main plant derived precursor for eicosapentaenoic acid (EPA), docosapentaenoic acid (DPA) and docosahexaenoic acid (DHA), yet conversion efficiency is limited and influenced by several dietary and metabolic factors. Therefore, the NuEva Study aimed to investigate the impact of flaxseed oil on fatty acid profiles depending on age, sex, body mass index (BMI), dietary pattern (Western diet (omnivores), flexitarian, vegetarian, vegan), and status of relevant nutrients.

**Methods:**

The NuEva study is a prospective non-randomized intervention with parallel diet groups (Western diet (omnivores), flexitarian, vegetarian, vegan; *n* = 168), which includes nutrient-optimized menu plans (12 months) combined with flaxseed oil supplementation (3 g/d ALA for 9 months). Fatty acids were analyzed at baseline and repeatedly throughout the intervention period focusing on *n*-6 and *n*-3 PUFA in plasma and erythrocytes lipids. Furthermore, potential modulators of ALA conversion (age, sex, BMI, linoleic acid, arachidonic acid, and EPA status) were investigated.

**Results:**

In Western diet participants, erythrocyte *n*-6 PUFA increased by 5.5%, mainly due to arachidonic acid. In contrast, ALA (+22.5–38.4%), EPA (+27.3–40.7%), DPA (+27.2–40.7%) and DHA (+12.8–26.0%) increased significantly across all dietary patterns. Conversion efficiency was unaffected by sex, BMI, age, linoleic acid, or arachidonic acid, but individuals with low baseline EPA showed markedly greater increases in EPA (+62.9% vs. +12.9%), DPA (+41.9% vs. +22.3%), and DHA (+27.0% vs. +7.6%) compared to subjects with higher EPA status.

**Conclusion:**

In conclusion, flaxseed oil supplementation combined with a controlled diet effectively improves *n*-3 LCPUFA status irrespective of habitual diet. The extent of relative improvement was primarily determined by baseline EPA concentrations.

## 1 Introduction

In recent years, there has been a growing interest in plant-based diets, with a rising number of individuals in Germany adopting a vegetarian and vegan diet (1). This trend is motivated by ethical and environmental concerns, as well as the potential health benefits associated with vegetarian diets (2). Scientific studies indicate that vegetarian and vegan diets are beneficial for cardiovascular health (3–5). However, the restriction of animal foods, particularly in vegan diets, raises concerns about the adequacy of specific nutrients, among others the (LC) *n*-3 polyunsaturated fatty acids (PUFA), eicosapentaenoic acid (EPA, C20:5*n*-3), docosapentaenoic acid (DPA, C22:5*n*-3) and docosahexaenoic acid (DHA, C22:6*n*-3) (6). Fish and seafood contain high amounts of EPA, DPA and DHA (7). As these foods are excluded in vegetarian and vegan diets, ensuring adequate supply of *n*-3 LCPUFA becomes challenging. *n*-3 LCPUFA play a structural role in the cell membrane influencing brain structure, fluidity, perfusion and signal transduction (8, 9). The fatty acids are also precursors to eicosanoids and docosanoids, such as prostaglandins, leukotrienes, thromboxanes, as well as resolvins and maresins, posing range of anti-inflammatory effects (10). An adequate supply with *n*-3 LCPUFA is important for cardiovascular as well as cognitive health and brain development (8, 11). Pregnant women following a vegetarian or vegan diet are especially vulnerable to insufficient DHA supply, as their status is often low, resulting in reduced amounts of DHA in breast milk and lower concentrations in erythrocyte phospholipids of their infants (12). In addition, epidemiological studies indeed revealed a lower risk and mortality for ischemic heart disease and type 2 diabetes for vegetarians and vegans but observed a higher risk for hemorrhagic stroke (13) and higher mortality due to neurological diseases, including stroke, dementia, and Parkinson’s disease in older adults following plant-based diets (14). These observations have been linked to lower *n*-3 LCPUFA levels, highlighting the importance of monitoring and improving the *n*-3 LCPUFA status.

To prevent cardiovascular disease, international organizations recommend a daily intake of EPA and DHA of 250–500 mg/d (15–17). However, fish consumption in Germany remains below recommended amounts (1–2 times per week), resulting in suboptimal EPA and DHA intakes (18, 19). Although EPA, DPA and DHA can be synthesized endogenously from the essential precursor *α*-linolenic acid (ALA, C18:3*n*-3) using desaturase and elongase steps (20), this conversion is limited and influenced by multiple factors, such as sex, age, genetics and individual metabolic conditions (21). Furthermore, linoleic acid (LA, C18:2*n*-6), an essential *n*-6 PUFA abundant in plant-based diets, may reduce the conversion efficiency of ALA into *n*-3 LCPUFA as it competes with ALA for the enzymes that catalyze the conversion of LA into the LC *n*-6 PUFA arachidonic acid (ARA, C20:4*n*-6) (22).

Considering the rising trend of individuals opting for a vegetarian or vegan diet, an efficient conversion of ALA into *n*-3 LCPUFA remains an important area of future research. To address this the NuEva study investigated whether plant-based *n*-3 PUFA from flaxseed oil can sufficiently support *n*-3 LCPUFA status in individuals following omnivorous, flexitarian, vegetarian and vegan diets. The study is of relevance because it included a high proportion of women of childbearing age (61%). The controlled dietary interventions based on energy- and nutrient-optimized menu plans which were provided depending on dietary pattern over 12 months. A regular intake of flaxseed oil was embedded, corresponding to an ALA intake of approximately 3 g/day over the last 9 months of the 12-month intervention period. Fatty acid profiles plasma and in erythrocyte lipids were measured every 3 months. The objective of this investigation was to examine the changes in EPA, DPA and DHA concentrations over the course of the study to clarify whether plant-based *n*-3 PUFA sources can increase *n*-3 LCPUFA status depending on dietary patterns. Furthermore, the impact of influencing factors on the conversion of ALA to its LC metabolites was investigated.

## 2 Materials and methods

### 2.1 Participants

**Figure 1:**
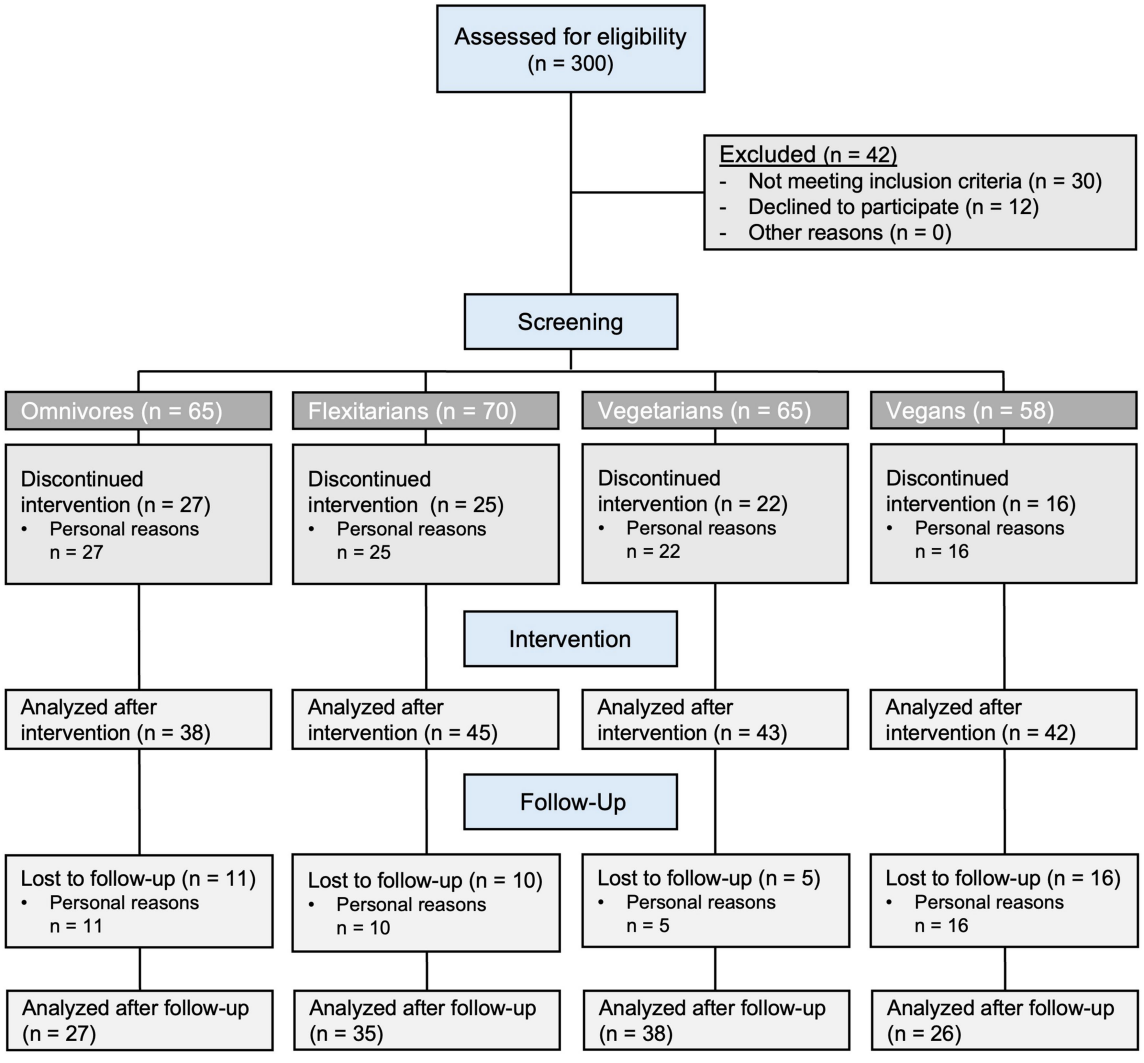
Flow diagram of the selection of NuEva study participants. A total of 300 subjects were enrolled in the study. A total of 42 subjects were excluded from the study either because they did not meet the inclusion criteria or because they declined to participate. The participants were divided into four groups (omnivores, flexitarians, vegetarians, and vegans) based on their dietary habits. Ninety nine subjects discontinued the intervention or did not adhere to every check-up for personal reasons (such as pregnancy, illness, compliance, expenditure of time), leaving 128 subjects for analysis of the intervention. In addition, 42 subjects were lost to follow-up, primarily due to the impact of the SARS-CoV-2 pandemic.

During the summer and autumn of 2018, healthy men and women aged 18 to under 70 years, who had followed an omnivorous, flexitarian, vegetarian, or vegan diet for at least 1 year prior to enrollment, were recruited for the study. Participants’ adherence to one of the four dietary patterns was confirmed through an interview before enrollment and verified by a dietary protocol before their first study visit. The omnivorous diet was similar to a typical Western diet, characterized by daily consumption of meat and sausages, while the flexitarian diet included only occasional meat and sausage consumption (once or twice per week). Fish consumption was not limited in both groups. Vegetarians were defined as individuals who consumed dairy products and eggs along with plant-based foods, while vegans were defined as those who consumed only foods of plant origin. Additional exclusion criteria have been published in the respective study protocol (23). In the present investigation, 38 omnivores, 45 flexitarians, 43 vegetarians, and 42 vegans who completed all four blood samplings during the intervention phase were included. Due to the SARS-CoV-2 pandemic, some participants were uncertain and therefore did not participate in the examinations after 12 and 24 months. A total of 27 Western diet subjects, 35 flexitarians, 38 vegetarians and 26 vegans completed the follow-up after 24 months and were included in the corresponding statistics for this additional time point (Figure 1).

### 2.2 Study design and diets

The study was designed as a prospective, non-randomized, monocentric intervention study in parallel design with four groups (omnivore, flexitarian, vegetarian, and vegan diets). The NuEva study aimed to assess the effects of common dietary patterns on health and disease risk, with a focus on improving nutritional behavior through three main strategies: (i) personalized menu plans tailored to individual needs, (ii) regular nutritional counseling sessions including feedback and progress tracking, and (iii) the use of incentive strategies. The menu plans adhered to the characteristics of each dietary pattern and were further defined by the following criteria:

- Appropriate distribution of energy from carbohydrates, protein, and fats according to the guidelines of the German Society of Nutrition (24).
- Defined limits for saturated fatty acids (SFA, <10% of daily energy), monounsaturated fatty acids (MUFA, >10% of daily energy), polyunsaturated fatty acids (PUFA, ~10% of daily energy), and at least 2 g of ALA per day, typically from 5 to 10 mL of flaxseed oil daily between study months 3 and 12.
- Encouraged intake of vegetables, fruits, and cereals.
- A dietary fiber intake of over 40 g per day based on the MoKaRi concept (25).
- Reduced consumption of salt (maximum 6 g per day) and sugar (maximum 50 g per day).
- Decreased intake of highly processed, calorie-dense, low-nutrient foods (e.g., fast food and convenience products).
- Optimized intake of vitamins, minerals, and trace elements through commonly available foods, considering the seasonal availability of vegetables and fruits.

The menu plans were generated using the PRODI software (version 6.4, Nutri-Science, Stuttgart, Germany). The plans were adjusted to each participant’s specific energy needs, considering age, gender, and physical activity levels. The menu plans included up to five meals per day, divided into breakfast, lunch, dinner, and up to two snacks. For each meal, comprehensive data on the specific food items and quantities were given. In addition, recipes for meals requiring preparation were also included. To enhance compliance, each participant was provided with flaxseed oil. The fatty acid composition of the provided oils was tested regularly (every 4–6 weeks) in the laboratory to monitor variations in ALA content across the batches. Throughout the study period, the fatty acid composition remained comparable (Table 1).

**Table 1:** Average fatty acid distribution in flaxseed oil measured over the study period (*n* = 40) expressed as percentage of fatty acid methyl esters (% FAME).

C16:0	C18:0	C18:1*n*-9	C18:2*n*-6	C18:3*n*-3	ΣSFA	ΣMUFA	ΣPUFA
4.8 ± 0.1 (4.4–4.9)	2.8 ± 0.3 (2.6–4.2)	15.4 ± 0.5 (14.7–16.7)	14.0 ± 0.4 (13.5–15.0)	61.4 ± 0.6 (60.1–62.1)	7.7 ± 0.3 (7.4–9.2)	16.1 ± 0.5 (15.4–17.4)	75.4 ± 0.5 (73.9–76.5)

Data is shown as mean ± standard deviation and (minimum – maximum); SFA = saturated fatty acids, MUFA = monounsaturated fatty acids, PUFA = polyunsaturated fatty acids.

The NuEva study began with a run-in phase to document the dietary habits through a five-day dietary protocol. To assess nutritional and health status, a single blood draw along with fecal and 24-h urine samples were collected during the screening (baseline) phase. During the 12-month intervention phase, participants were provided with nutrient-optimized daily menu plans. Flaxseed oil was added from the third month on. Checkups, including anthropometric measurements, pulse and blood pressure assessments, and blood sampling, were conducted every 3 months. Following the 12-month intervention, participants entered a 12-month follow-up period without nutritional coaching, and additional blood samples were taken afterwards. More details about the study design can be found in the published study protocol (23).

### 2.3 Laboratory parameters

#### 2.3.1 Sample collection

Blood samples were collected by venipuncture after at least 12 h overnight fasting. For separation of plasma, blood was collected in lithium-heparin monovettes at room temperature, and centrifuged for 10 min (2,762 × *g*, 4 °C). After plasma was removed, the residual erythrocytes were washed with an equal volume of physiological sodium chloride solution (0.9%) and centrifuged for 10 min (1,300 × *g*, 4 °C). This step was repeated until the supernatant was clear. All samples were stored at −80 °C until analysis.

#### 2.3.2 Lipid extraction and fatty acid analysis

Plasma and erythrocyte lipids were extracted following the protocols of Folch, Bligh, and Dyer et al. (26, 27), utilizing a methanol/chloroform/water mixture in a 2:1:1 ratio (v/v/v). The extracted lipids were saponified and methylated using NaOCH_3_ (Carl Roth, Karlsruhe, Germany) and BF_3_ (Sigma-Aldrich, Steinheim, Germany) as previously detailed (28). To purify the resulting products post-methylation, thin-layer chromatography was employed on silica gel aluminum plates (Merck, Darmstadt, Germany) with hexane:diethyl ether:acetic acid (85:15:0.2, v/v/v). Fatty acid methyl esters (FAME) were analyzed using a gas chromatograph (GC-17 V3; Shimadzu, Duisburg, Germany) equipped with a flame ionization detector. Various standards were applied for fatty acid peak identification (No. 463, 674 (NU-CHEK PREP; INC., US), BR2, BR4, and ME 93) (Larodan Fine Chemicals, Larodan, Sweden) Menhaden (Sigma Aldrich, Steinheim, Germany). Quantification of each FAME was achieved using LabSolution software (LabSolution LC/GC release 5.92, Shimadzu), and the concentrations of individual fatty acids were expressed as a percentage of the total area of all fatty acid peaks (% FAME).

For calculations of the sum of *n*-3 PUFA, the following *n*-3 PUFA were included: ALA (C18:3*n*-3), eicosatetraenoic acid (C20:4*n*-3), EPA (C20:5*n*-3), DPA (C22:5*n*-3) and DHA (C22:6*n*-3). The sum of *n*-6 PUFA comprises the following fatty acids: LA (C18:2*n*-6), *γ*-linolenic acid (GLA, C18:3*n*-6), C20:2*n*-6, C20:3*n*-6, ARA (C20:4*n*-6), C22:4*n*-6 and C22:5*n*-6. The *n*-3 index was calculated by using the sum of EPA and DHA in erythrocyte lipids. The *n*-3 LCPUFA correspond to the sum of EPA, DPA and DHA. Further, %*n-*6 and %*n*-3 in highly unsaturated fatty acids (HUFA), which comprise fatty acids with three or more double bonds in their carbon chains, were calculated according to Equations 1, 2, respectively.


$$\%n-6\;\text{in HUFA}:100\times \frac{\begin{array}{l} C20:3n-6+\mathrm{ARA}+C22:\\ 4n-6+C22:5n-6 \end{array}}{\begin{array}{l} C20:3n-6+\mathrm{ARA}+C22:\\ 4n-6+C22:5n-6+C20:\\ 3n-3+C20:4n-3+\\ \mathrm{EPA}+\mathrm{DPA}+\mathrm{DHA} \end{array}}\;\;$$ (1)

$$\%n-3\;\text{in HUFA}:100\times \frac{\begin{array}{l} C20:3n-3+C20:\\ 4n-3+\mathrm{EPA}+\mathrm{DPA}+\mathrm{DHA} \end{array}}{\begin{array}{l} C20:3n-6+\mathrm{ARA}+C22:\\ 4n-6+C22:5n-6+C20:\\ 3n-3+C20:4n-3+\\ \mathrm{EPA}+\mathrm{DPA}+\mathrm{DHA} \end{array}}$$ (2)

Phenotypic markers of desaturase activity were used as surrogate measures of tissue desaturase activities based on the ratios of relevant product and precursor fatty acids, as follows: C18:1*n*-9/18:0 (Δ9-desaturases, D9D), C20:4*n-*6/C20:3*n-*6 (Δ5-desaturase, D5D), C20:5*n-*3/C20:4*n-*3 (D5D), C20:3*n-*6/C20:2*n-*6 (Δ6-desaturase, D6D), and C20:5*n-*3/C18:3*n*-3 (D6D).

### 2.4 Statistical analyses

The analysis of fatty acids in plasma and erythrocyte lipids was conducted as an exploratory aim. Accordingly, the sample size was calculated based on the primary outcome parameters of the trial. A power calculation was performed for the low-density lipoprotein (LDL) cholesterol/high-density lipoprotein cholesterol ratio, based on data from Li et al. (29). A sample size of 44 participants per group was calculated to achieve an 80% power. In anticipation of a 25% dropout rate, the aim was to recruit a minimum of 55 participants per group. The power analysis for the NuEva study was conducted using G*Power (The G*Power Team, Düsseldorf, Germany, version 3.1.9.2). The statistical analysis was performed by SPSS statistics (IBM Germany, Ehningen, Germany, Version 29.0.0.0). In this publication, the period from the third to the 12th month was considered, as the focus was on the conversion of ALA through the supplementation of flaxseed oil. Only data from subjects who attended every study appointment of the intervention phase were included for statistical testing. The Shapiro–Wilk test was performed to examine whether the data followed a normal distribution. Differences within groups while comparing each point in time were assessed using ANOVA for repeated measurements for normally distributed variables or the Friedmann test if data were not normally distributed. Differences between the dietary patterns (including all tests comparing changes from baseline between groups), were assessed using ordinary one-way ANOVA for parametric variables or Kruskall-Wallis test for non-parametric variables. As a post-hoc test, Fisher’s least significant difference test was used, and all calculated *p*-values were adjusted manually using the Benjamini–Hochberg procedure. For categorical variables Chi^2^-test was applied. We divided our study collective according to sex, body mass index (BMI; <22.42 vs. ≥22.42), age (<28 vs. ≥28), LA status (<12.59 vs. ≥12.59% FAME), ARA status (<13.29 vs. ≥13.29% FAME) and EPA status at baseline (<0.58% FAME vs. ≥0.58% FAME) to evaluate the influence of these factors on the conversion of ALA into its LC metabolites. The limits represent the cut-off values of each 50% of our participants (median). In addition, follow-up data were analyzed (12 months after the end of the intervention). Only data from subjects who had blood samples taken at the end of the intervention were considered. The follow-up data were compared within the groups with the end of the intervention using a paired *t*-test if they were normally distributed. Otherwise, the Mann–Whitney *U* test was performed. Again, differences between the dietary patterns (including all tests comparing changes from intervention between groups), were assessed using ordinary one-way ANOVA for parametric variables or Kruskall-Wallis test for non-parametric variables (using Benjamini-Hochberg correction). Normally distributed data were presented as mean ± standard deviation (SD), whereas skewed data were presented as median / interquartile range (IQR). Statistical significance is referred to *p*-values smaller than 0.05. To visualize the differences in *n*-3 and *n*-6 PUFA concentrations between the diet groups at each time point and the changes within each group throughout the follow-up, we fitted linear mixed models with each lipid as dependent variable and participant as random effect. The fixed effects included sex, age, BMI, diet, and time. We modelled an interaction of diet and time with a cubic polynomial term to allow non-linear trajectories for each diet over time. The box plots were generated using R studio (Public Benefit Corporation, Boston, Massachusetts, USA, version 2022.12.0) running R software (The R Foundation for Statistical Computing, Vienna, Austria, version 4.2.3).

## 3 Results

### 3.1 Changes in fatty acid profiles during the intervention

In the NuEva study, fatty acid profiles were analyzed in both plasma and erythrocyte lipids over a period of 9 months at 3-month intervals. The main emphasis of this analysis focused on changes in *n*-6 and *n*-3 PUFA in erythrocyte lipids. For SFA and MUFA, only marginal changes were observed, with comparable percentual changes from the start of intervention (%CSI) across the four dietary groups. Detailed data on changes of SFA and MUFA in erythrocyte lipids are provided in Supplementary Table S1 (intervention) and Supplementary Table S2 (follow-up).

Regarding total *n-*6 levels, only the Western diet group exhibited an increase of 5.5% in erythrocyte lipids during the intervention period (*p* < 0.01), with no significant differences in %CSI between the groups. At both the start and end of the intervention, vegans had the highest proportions of total *n-*6 PUFA in erythrocyte lipids (*p* ≤ 0.04). This was mainly due to higher LA levels compared to other groups throughout the study (*p* < 0.001), with increasing exclusion of animal foods in general resulting in higher LA proportions at month 12 (VN > VG > Flex > WD, *p* < 0.05). The LA levels showed no significant change throughout the study in any group. GLA concentration in erythrocytes increased about 16.2% in flexitarians over the study period (*p* < 0.001). Overall, GLA concentrations were comparable between the groups. Vegans had an increase of 8.0% in erythrocytes concentrations of C20:2*n-*6 (*p* < 0.001) and Vegans exhibited the highest levels of C20:2*n-*6 throughout the intervention (*p* < 0.001). Dihomo-*γ*-linolenic acid (C20:3*n*-6) concentrations slightly increased in all groups over time (*p* ≤ 0.04), with no significant differences in the %CSI between the groups. Vegetarians had the highest concentrations of dihomo-γ-linolenic acid at the start of the intervention, while vegans exhibited the highest concentrations at the end of the intervention compared to flexitarians (both *p* < 0.001). ARA concentrations increased in both omnivores groups over time, with the %CSI in the Western diet group being significantly higher (12.02%) compared to the other groups (*p* < 0.001). Western diet subjects had higher ARA concentration in erythrocytes at the end of the intervention compared to the other groups, while vegans exhibited the lowest ARA values throughout the intervention (both *p* < 0.001). The erythrocyte concentrations of adrenic acid (C22:4*n-*6) did not change significantly in any group during the intervention. Overall, no group differences were evident at month 12. Osbond acid (C22:5*n-*6) remained stable within the groups in erythrocytes, with vegans exhibiting the lowest values compared to the other groups (*p* ≤ 0.001). Vegans had the lowest values in erythrocytes lipids compared to the other four groups (*p* < 0.01) (Table 2).

The total *n-*3 concentrations increased significantly in all groups over the study period ranging from 19.1 –33.5% in erythrocyte lipids (*p* < 0.001). However, there were no significant differences in the %CSI between the groups. Vegans had lower concentrations in erythrocytes than omnivorous and vegetarian participants at month 12 (*p* ≤ 0.04). In addition, vegetarians had lower levels than Western diet participants (*p* < 0.001) but higher levels than vegans (*p* < 0.05). The consumption of flaxseed oil as part of the intervention phase increased the levels of ALA in the erythrocyte lipids regardless of the dietary pattern (*p* ≤ 0.02). However, an increase from month 3 to 12 for the ALA concentrations was only significant in the Western diet group (38.4%), flexitarians (28.2%), and vegetarians (23.5%) (*p* < 0.01). Furthermore, the %CSI did not differ between the four dietary patterns. At the beginning of the intervention the Western diet group had the lowest concentrations of ALA in erythrocyte lipids (*p* < 0.001), although this difference was no longer observed at month 12. Eicosatetraenoic acid (C20:4*n*-3) concentrations increased significantly in both omnivores groups (34.7–35.9%) (*p* ≤ 0.03). Differences in the %CSI were not significant. Initially, vegans had lower erythrocyte eicosatetraenoic acid concentrations compared to vegetarians (*p* = 0.02), and after the intervention, their concentrations remained lower compared to the Western diet group (*p* < 0.001) and flexitarians (*p* = 0.02). EPA and DPA levels increased in all groups over the study period (*p* ≤ 0.01), with vegans showing the greatest %CSI of 41.0 and 40.7%. Vegans had lower EPA concentrations compared to other groups throughout the intervention (*p* ≤ 0.01), while DPA did not differ between the four dietary patterns. DHA concentration increased significantly in erythrocyte lipids (12.8 –26.0%) in all groups (*p* ≤ 0.03), with vegans and vegetarians having lower concentrations compared to omnivorous subjects throughout the intervention phase (*p* < 0.001). Therefore, the *n-*3 index and the sum of *n*-3 LCPUFA in erythrocytes also increased significantly in all groups (*p* ≤ 0.04), with no significant differences in %CSI between the groups. Regarding the proportion of *n-*6 in HUFA decreased in all groups over the study period (−3.3 – 4.7%) (*p* < 0.001), with vegans consistently having the highest levels. Conversely, the proportion of *n-*3 in HUFA increased in all groups (10.0 –17.4%) (*p* < 0.001), with vegans having the highest change but consistently the lowest absolute concentration (Table 2).

**Table 2:** Polyunsaturated fatty acid profile (% FAME) in erythrocyte lipids during the intervention according to diet group.

	t	WD (27 w, 11 m)	⋄	Δ	Flex (37 w, 8 m)	⋄	Δ	VG (30 w, 13 m)	⋄	Δ	VN (28 w, 14 m)	⋄	Δ
*n*-6 PUFA													
C18:2*n*-6	3	12.13/2.03 11.85 ± 1.49	a,b	a	12.15/2.88	a	a,b	12.61/2.32 12.70 ± 2.14	a	b	14.27/2.65	a	c
	6	11.61/1.92 11.96/1.59	a	a*	12.24/1.93 12.48 ± 1.25	a	a*	13.24 ± 1.75	a	b	14.40/2.04 14.46 ± 1.45	a	c
	9	11.51/1.49 11.39 ± 1.24	b	a	12.05/1.64	a	a,b	12.42/1.82 12.83 ± 1.75	a	b	13.78/2.13	a	c
	12	11.49/1.91 11.53 ± 1.39	b	a	12.19/1.58 12.18 ± 1.13	a	b	12.87 ± 1.55	a	c	14.36/2.12 14.24 ± 1.42	a	d
	%	−2.44/12.94		a	1.53/16.55		a	−2.09/10.18		a	−2.26/9.84		a
C18:3*n*-6	3	0.04/0.01	a	a,b	0.03/0.02	a	a	0.04/ 0.02	a	b	0.03/0.01	a	a,b
	6	0.04/0.02	b	a	0.04/0.02	b	a	0.04/0.03	a	a	0.04/0.02	a	a
	9	0.04/0.02	a,b	a	0.04/0.01	b	a	0.04/0.02	a	a	0.04/0.02	a	a
	12	0.04/0.02	a,b	a	0.04/0.02	b	a	0.05/0.02	a	a	0.04/0.02	a	a
	%	10.98/45.90		a	16.23/42.58		a	7.69/48.20		a	9.22/47.20		a
C20:2*n*-6	3	0.20/0.06	a	a	0.24/0.09	a	b	0.23/0.07	a	b	0.36/0.18	a	c
	6	0.20/0.05	a	a	0.23/0.05	a	b	0.25/0.05	a	b	0.41/0.13	b	c
	9	0.19/0.03	b	a	0.23/0.06	a	b	0.23/0.05	b	b	0.38/0.11	a	c
	12	0.21/0.04	a	a	0.22/0.03	a	b	0.25/0.07	a	b	0.40/0.14	b	c
	%	3.81/20.98		a,b	−1.71/23.55		a	0.46/26.01		a,b	8.03/24.69		b
C20:3*n*-6	3	1.35/0.37	a	a,b	1.36/0.40	a,b	a	1.53/0.41	a	b	1.45/0.43	a,b	a,b
	6	1.31/0.42	a,b	a	1.39/0.43	a,b	a	1.46/0.41	a	a	1.60/0.40	a,b	a
	9	1.24/0.39	a	a	1.35/0.35	a	a	1.44/0.48	a	a	1.50/0.39	a	a
	12	1.48/0.34	b	a,b	1.38/0.32	b	a	1.60/0.51	a	a,b	1.62/0.55	b	b
	%	7.88/27.29		a	5.00/25.68		a	1.58/25.23		a	9.22/26.85		a
C20:4*n*-6	3	13.63/1.79	a	a	13.20/2.72	a	a	13.41/2.08	a,b	a	13.02/2.05	a,b	a
	6	14.15/2.66	b	a	14.15/2.73	b	a,b	13.75/2.98	a	a,b	13.20/2.09	a	b
	9	13.95/1.57	a	a	13.47/2.08	a	a,b	12.96/2.04	b	b,c	12.86/1.80	b	c
	12	15.37/3.24	b	a	13.54/1.93	a,b	b	13.06/1.61	a,b	b	13.63/2.09	a	b
	%	12.02/16.35		a	4.10/17.65		a,b	0.45/24.90		b	5.55/16.77		a
C22:4*n*-6	3	2.52/0.83	a,b	a	2.56/0.90	a	a	2.71/0.80	a	a,b	2.96/0.88	a	b
	6	2.54/0.82 2.66 ± 0.64	a	a	2.74/0.81 2.79 ± 0.60	a	a	2.86/0.89 2.82 ± 0.70	a	a	3.06/0.78 3.01 ± 0.67	a	a
	9	2.58/0.49	a,b	a	2.65/0.66	a	a	2.84/1.04	a	a	2.84/0.75	a	a
	12	3.06/1.21	b	a	2.73/0.66	a	a	2.82/0.91	a	a	2.90/0.94	a	a
	%	11.39/40.05		a	3.87/27.09		a	5.03/15.43		a	3.83/24.66		a
C22:5*n*-6	3	0.36/0.15	a	a	0.34/0.16	a,b	a	0.34/0.17	a	a	0.33/0.12	a	a
	6	0.35/0.16	a	a	0.35/0.20	a	a	0.36/0.14	a	a	0.31/0.12	a	b
	9	0.37/0.13	a	a	0.34/0.15	a,b	a	0.35/0.13	a	a	0.29/0.11	a	b
	12	0.37/0.23	a	a	0.32/0.19	b	a	0.34/0.16	a	a	0.29/0.11	a	b
	%	12.62/36.47		a	−2.24/39.35		a	−0.35/31.97		a	−2.84/29.57		a
Σ *n*-6	3	29.86/2.36	a	a	30.54/3.89	a	a,b	31.13/2.62	a,b	b	32.45/3.69	a	c
	6	31.15/3.16	a	a	31.36/2.16	b	a	32.69/3.71	a	a,b	33.42/3.07	a	b
	9	29.92/2.37 29.96 ± 1.68	b	a	30.36/1.93 20.23 ± 1.56	a	a,b	30.86/2.31 30.85 ± 1.67	b	b,c	31.85/2.69 31.54 ± 1.93	b	c
	12	31.92/2.79	b	a,c	30.22/2.64	a,b	b	31.66/3.06	a,b	a	32.83/3.66	a	c
	%	5.51/12.58		a	1.02/12.70		a	1.23/12.35		a	3.38/8.75		a
*n*-3 PUFA													
C18:3*n*-3	3	0.17/0.09	a	a	0.18/0.12	a	a,b	0.19/0.15	a	a,b	0.21/0.17	a	b
	6	0.25/0.17	b	a	0.25/0.18	b	a	0.29/0.15	b	a	0.27/0.18	a,b	a
	9	0.24/0.14	b	a	0.26/0.22	b	a,b	0.25/0.18	b	a,b	0.30/0.15	b	b
	12	0.23/0.12	b	a	0.26/0.18	b	a	0.28/0.21	b	a	0.28/0.19	a,b	a
	%	38.43/77.65		a	28.22/116.82		a	23.54/75.19		a	22.49/84.86		a
C20:4*n*-3	3	0.05/0.03	a	a,b	0.05/0.03	a	a,b	0.06/0.04	a,b	a	0.05/0.05	a	b
	6	0.07/0.03	b	a	0.06/0.05	a	b	0.06/0.04	a	b	0.05/0.04	a	b
	9	0.08/0.02	b	a	0.08/0.04	b	a	0.07/0.04	b	a	0.05/0.03	a	b
	12	0.08/0.05	b	a	0.07/0.04	b	a	0.07/0.05	a,b	a	0.06/0.04	a	b
	%	34.69/64.22		a	35.94/95.59		a	−10.98/81.30		a	9.38/89.97		a
C20:5*n*-3	3	0.75/0.49	a	a	0.63/0.54 0.70 ± 9.31	a	a	0.66/0.44	a	a	0.35/0.39	a	b
	6	0.79/0.47	a	a	0.71/0.42 0.76 ± 0.28	a	a,b	0.57/0.38	a	b,c	0.46/0.39	a	c
	9	0.85/0.44	b	a	0.89/0.49 0.90 ± 0.34	b	a	0.70/0.50	b	a,b	0.53/0.48	b	b
	12	1.02/0.68	b	a	0.94/0.55 0.94 ± 0.36	b	a,b	0.72/0.50	b	b	0.61/0.45	b	c
	%	29.77/72.44		a	27.26/78.32		a	29.94/80.74		a	40.69/82.29		a
C22:5*n*-3	3	2.07/0.57	a	a	2.07/0.45 2.07 ± 0.53	a	a	2.33/0.86 2.23 ± 0.55	a	a	1.96/0.66	a	a
	6	2.13/0.44	a	a	2.28/0.52 2.31 ± 0.36	b	a	2.37/0.86 2.42 ± 0.74	b	a	2.27/0.83	b	a
	9	2.47/0.51	b	a	2.41/0.55 2.51 ± 0.45	c	a	2.67/0.75 2.70 ± 0.49	c	a	2.49/0.55	c	a
	12	2.73/0.82	b	a	2.52/0.63 2.59 ± 0.50	c	a	2.79/0.87 2.88 ± 0.59	d	a	2.54/1.01	c	a
	%	39.56/48.54		a	27.22/37.41		a	32.50/30.76		a	40.74/34.77		a
C22:6*n*-3	3	3.69/1.64 3.66 ± 0.97	a	a	3.32/1.60 3.28 ± 1.05	a	a,b	2.73/1.19	a	b	2.04/1.00	a	c
	6	3.78/1.40 3.94 ± 0.88	a,b	a	3.75/1.47 3.90 ± 1.00	b	a	2.84/1.20	a,b	b	2.19/1.18	a	b
	9	3.96/1.11 4.07 ± 0.89	b	a	3.86/1.46 4.01 ± 1.10	b	a	3.00/1.27	b,c	b	2.46/1.30	b	b
	12	4.41/1.10 4.42 ± 0.96	c	a	3.98/1.37 4.09 ± 1.00	b	a	3.14/1.37	c	b	2.67/1.09	b	b
	%	25.99/49.77		a	12.84/39.08		a	17.49/31.99		a	20.75/45.37		a
*n*-3 index	3	4.48/1.75 4.45 ± 1.22	a	a	3.92/2.02	a	a,b	3.40/1.34	a	b	2.47/1.19	a	c
	6	4.76/1.74 4.80 ± 1.10	a,b	a	4.53/1.77	b	a	3.46/1.36	a,b	b	2.73/1.20	b	b
	9	4.83/1.43 4.99 ± 1.05	b	a	4.57/1.75	b	a	3.76/1.48	b,c	b	2.97/1.58	c	b
	12	5.42/1.46 5.42 ± 1.16	c	a	5.00/1.68	b	a	4.07/1.34	c	b	3.17/ 1.39	c	b
	%	28.65/48.75		a	20.76/3.41		a	16.08/35.98		a	26.67/44.00		a
*n*-3 LCPUFA	3	6.46/2.04 6.55 ± 1.47	a	a	6.19/1.88	a	a	5.76/1.69 5.82 ± 1.23	a	a	4.52/1.70	a	b
	6	7.02 ± 1.25	a	a	6.96/1.91 6.97 ± 1.39	b	a	5.80 ± 2.06	a	b	5.09/2.14 5.28 ± 1.56	b	b
	9	7.24/1.56 7.43/1.21	b	a	7.13/2.04	c	a	6.42/1.46 6.51 ± 1.28	b	b	5.69/1.95	c	b
	12	8.16/2.27 8.19 ± 1.56	c	a	7.29/2.19	c	a	6.84/2.05 6.97 ± 1.37	c	b	6.24/2.15	c	b
	%	34.70/43.81		a	22.35/34.77		a	20.25/31.70		a	25.93/43.24		a
Σ *n*-3	3	6.80/2.17	a	a	6.49/2.16	a	a	6.17/1.96 6.21 ± 1.28	a	a	4.92/2.17	a	a
	6	7.25/1.81 7.35 ± 1.26	a	a	7.31/1.94 7.31 ± 1.41	b	a	6.31 ± 1.77	a	b	5.43/2.21 5.65 ± 1.63	b	b
	9	7.56/1.62	b	a	7.55/2.00	c	a	6.76/1.58 6.90 ± 1.38	b	b	6.04/2.23 6.30 ± 1.51	c	b
	12	8.49/2.15	c	a	7.62/2.43	c	a,b	7.18/2.09	c	b	6.66/2.30	c	c
	%	33.50/43.62		a	20.78/33.50		a	19.08/35.53		a	22.88/42.97		a
Σ PUFA	3	37.40/2.17	a	a	37.29/2.84	a	a	37.47/2.42	a	a	37.65/2.45	a	a
	6	38.25/1.63	a	a	38.74/1.87	b	a	39.05/3.34	b	a	38.59/3.46	b,c	a
	9	37.65/2.27	a	a	37.89/1.90	a,b	a	37.91/2.56	a	a	37.51/2.20	a,b	a
	12	41.36/4.03	b	a	38.36/2.51	a,b	b	38.87/3.24	b	b	39.24/3.86	c	a,b
	%	9.37/15.49		a	3.21/10.97		a	5.42/12.00		a	6.17/9.47		a
%*n*-6^1^	3	72.62/8.24 72.90 ± 5.00	a	a	73.37/7.51 73.11 ± 4.78	a	a	75.34/5.41	a	a	79.71/7.67	a	a
	6	72.96/6.53 72.82 ± 4.13	a	a	71.83/7.43 72.67 ± 4.57	a	a	76.23/6.54	a	b	78.07/6.20	a	b
	9	71.27/6.53 70.96 ± 4.21	b	a	71.26/8.64 70.47 ± 4.91	b	a	73.18/6.10	b	a,b	76.01/8.05	b	b
	12	70.29 /6.00 70.73 ± 4.07	b	a	70.45/5.26 70.39 ± 4.74	b	a	70.83/6.30	b	a,b	74.59/7.81	b	b
	%	−4.49/6.27		a	−3.07/7.10		a	−3.79/4.88		a	−3.65/6.16		a
%*n*-3^2^	3	27.38/8.24 27.10 ± 5.00	a	a	26.63/7.51 26.89 ± 4.78	a	a	24.66/5.41	a	a	20.29/7.67	a	b
	6	27.04/6.53 27.18 ± 4.11	a	a	28.17/7.43 27.33 ± 4.57	a	a	23.77/6.54	a	b	21.93/6.20	a	b
	9	28.73/6.53 29.04 ± 4.21	b	a	29.53 ± 4.91	b	a	26.82/6.10 27.07 ± 4.66	b	a,b	23.99/8.05 25.76 ± 6.43	b	b
	12	29.71/6.00 29.27 ± 4.07	b	a	29.55/5.26 29.61 ± 4.74	b	a	27.17/6.30	b	a,b	25.41/7.81	b	b
	%	13.97/18.57		a	9.14/19.05		a	12.29/16.65		a	15.97/23.77		a

Variable expressed as mean (±SD) and/or as median/IQR depending on the statistical test that was performed; ◊ comparison of time points within diet groups; Δ comparison between diet groups at individual time points; groups without a common letter are significantly different, *p* < 0.05; WD = omnivores, Flex = flexitarians, VG = vegetarians, VN = vegans, m = men, w = women, t = time point (month), % = percentual change from start of intervention, PUFA = polyunsaturated fatty acids, n = omega, LC = long-chain, HUFA = highly unsaturated fatty acids. ^1^Percentage of *n*-6 PUFA in fatty acids with three or more double bonds. ^2^Percentage of *n*-3 PUFA in fatty acids with three or more double bonds.

In the follow-up assessment total *n-*6 PUFA in erythrocytes increased in flexitarians and vegetarians (*p* ≤ 0.006), with vegetarians and vegans having higher concentrations at follow-up compared to omnivores (*p* ≤ 0.01). LA decreased (*p* ≤ 0.03) while dihomo-*γ*-linolenic acid concentrations increased in all groups (*p* ≤ 0.01). In erythrocytes, ARA concentrations increased in flexitarians (*p* < 0.01) and were still lower in vegetarians and vegans compared to both omnivores groups (*p* < 0.05). Adrenic acid and osbond acid concentrations increased universally (*p* = 0.001), with vegans still showing the lowest values at follow-up, compared to the other groups (*p* ≤ 0.02). The sum of *n-*3 PUFA in erythrocyte lipids increased at follow-up in the flexitarians (*p* < 0.01), whereas the other groups showed comparable values to the end of the intervention. Vegetarians and vegans had lower amounts of *n-*3 PUFA compared to the omnivorous groups (*p* ≤ 0.04). After the intervention, ALA levels decreased in all groups (*p* < 0.001) and vegetarians and vegans exhibited higher erythrocyte ALA concentrations compared to both omnivores groups at follow-up (*p* ≤ 0.03). Eicosatetraenoic acid concentrations decreased (*p* ≤ 0.02) and the values were comparable between the four dietary patterns. Regarding EPA, a decline in erythrocyte lipids was evident in all groups, but only significant for Western diet subjects and vegetarians (*p* < 0.001). At follow up, vegans still had the lowest values for erythrocyte and plasma EPA (*p* < 0.001). DPA levels increased in vegetarians in erythrocyte lipids (*p* < 0.001), while DHA levels and *n-*3 Index increased in flexitarians and vegans as well (*p* ≤ 0.04). The %CSI in vegans was about 20.5 and 19.2%, respectively, and differed significantly from the Western diet group (*p* = 0.02). Nevertheless, the levels of DHA in plasma lipids were still lower in vegetarians (both, *p* ≤ 0.04) and vegans (both, *p* < 0.001) compared to both omnivorous groups. The sum of erythrocyte *n*-3 LCPUFA only increased in flexitarians (*p* = 0.01) and vegetarians (*p* ≤ 0.04) and were lower in vegetarians and vegans compared to flexitarians and Western diet subjects (*p* < 0.05) (Supplementary Table S2).

**Figure 2:**
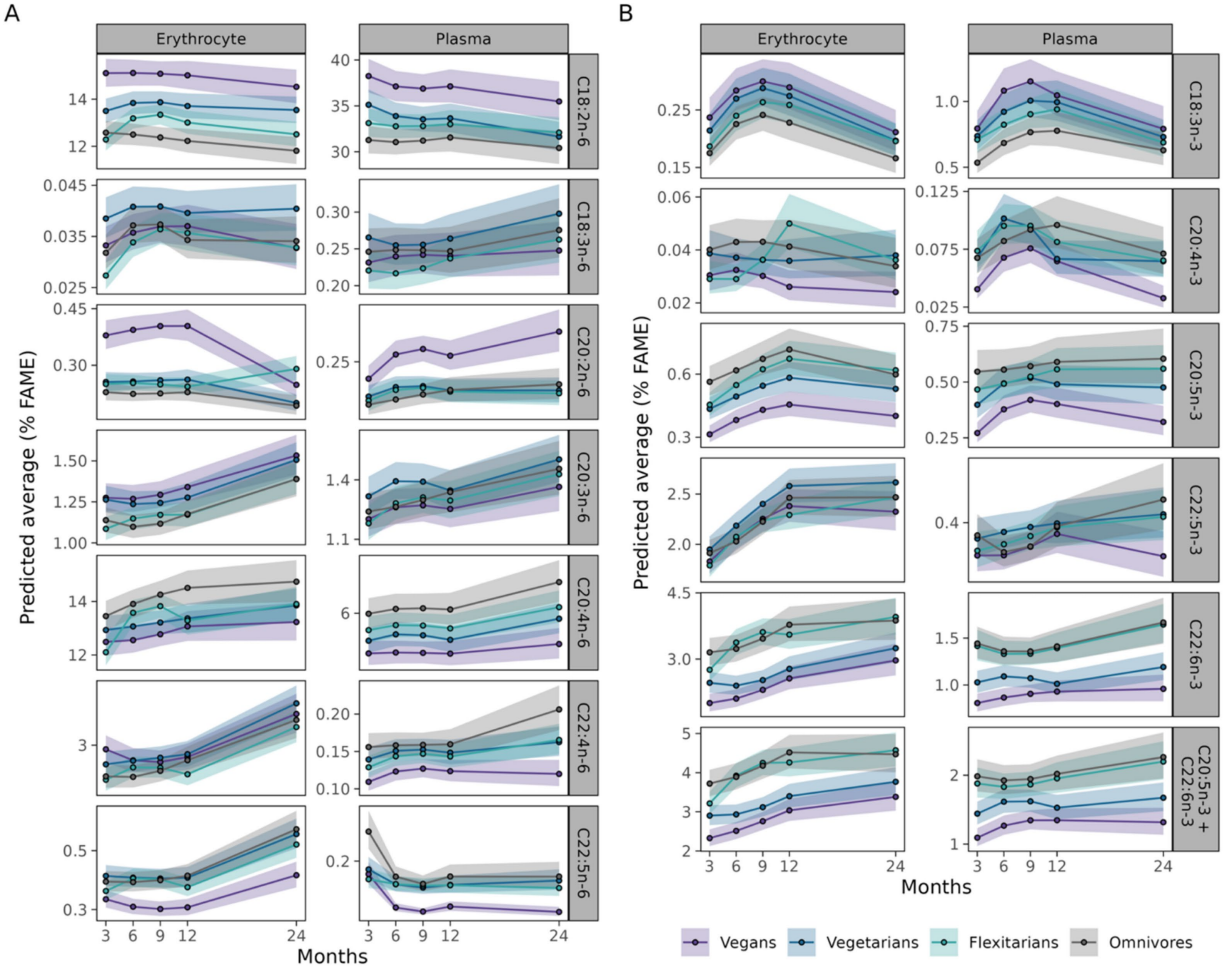
Comparison of selected *n-*6 PUFA **(A)** and *n*-3 PUFA **(B)** during the study according to dietary pattern. Fatty acid concentrations of Western diet subjects (Omnivores, grey line) flexitarians (green line), vegetarians (blue line), and vegans (lilac line) were determined in erythrocyte and plasma lipids as percentage of total fatty acid methyl ester (%FAME). The results are shown as predicted average with 95% confidence interval.

To comprehensively evaluate the effects of ALA supplementation on LC PUFA status, changes in the relevant fatty acids in plasma and erythrocyte lipids were compared. For *n*-6 PUFA, LA and GLA remained stable in plasma, with LA being consistently higher in vegans compared to the other groups across both matrices. C20:2*n*-6 was also highest in vegans for both erythrocyte and plasma lipids, remained stable throughout the intervention, but showed a non-significant decrease in erythrocytes and increase in plasma of vegan subjects. C20:3*n*-6 showed different patterns for the group differences between plasma and erythrocytes, with vegans having the highest concentrations in erythrocyte and lowest concentrations in plasma lipids. In all groups a slight increase was observed for both plasma and erythrocytes at the follow-up. Notably the observed changes in erythrocyte ARA concentrations for both omnivores groups were not evident in plasma lipids, which remained stable for all groups. The group differences in ARA concentrations were reflected in both plasma as well as erythrocyte lipids (Western diet > flexitarians > vegetarians > vegans). The increase in plasma adrenic acid concentrations at follow-up was comparable to that observed in erythrocyte lipids. However, the overall pattern of group differences differed between the two matrices, with vegans only showing lower plasma concentrations of adrenic acid. In accordance, vegans also exhibited the lowest osbond acid concentrations in both plasma and erythrocytes. While osbond acid levels increased in erythrocytes, plasma levels decreased in all groups by the follow-up (Figure 2A). Regarding *n*-3 PUFA, the changes in plasma and erythrocyte lipids were more comprehensive compared to *n*-6 PUFA. In accordance with erythrocyte lipids, plasma reflected the increase in ALA, EPA and DPA, but showed more fluctuations throughout the intervention. Furthermore, DHA remained stable in plasma, with only a slight increase in both omnivores groups at the follow-up, which contrasts the observed gradual increase in erythrocyte lipids over the course of the study. Across all time points, changes were more pronounced and consistent in erythrocytes. However, both matrices revealed clear group differences depending on dietary pattern, with vegans exhibiting the highest ALA, but lowest C20:4*n*-3, EPA, DPA and DHA concentrations (Figure 2B).

### 3.2 Changes in fatty acid ratios during the intervention

The *n-*6/*n-*3, ARA/EPA, ARA/DHA, and LA/ALA ratios all decreased significantly during the intervention period (*p* ≤ 0.03), with no differences in %CSI between groups. Initially, vegans had a higher *n-*6/*n-*3 ratio than the other groups (*p* < 0.001), which persisted throughout the study. At month 12, vegetarians also differed from Western diet subjects and flexitarians (*p* ≤ 0.03). The ARA/DHA ratio was higher in vegetarians and vegans (both *p* ≤ 0.02), while the ARA/EPA ratio was highest in vegans (*p* < 0.001). The LA/ALA ratio remained unaffected, but the ARA/LA ratio increased significantly in the Western diet group (*p* < 0.001), with the most pronounced change in Western diet subjects (16.0%) compared to vegetarians (1.2%; *p* = 0.01). Vegans had the lowest ARA/LA ratio at month 3 and throughout the study (*p* < 0.001), with vegetarians showing a lower ratio than Western diet subjects initially (*p* = 0.04), but not at the end of the intervention phase (Table 3).

**Table 3:** Comparison of fatty acid ratios in erythrocyte lipids during the intervention period according to diet group.

	*t*	WD (27 w, 11 m)	⋄	Δ	Flex (37 w, 8 m)	⋄	Δ	VG (30 w, 13 m)	⋄	Δ	VN (28 w, 14 m)	⋄	Δ
*n*-6/*n*-3	3	4.37/1.72	a	a	4.44/1.50 4.70 ± 1.07	a	a	5.18/1.69	a	a	6.66/3.01	a	b
	6	4.17/1.34	a	a	4.24/1.30 4.47 ± 0.97	a	a	5.35/1.81	a	b	6.11/2.62	a	b
	9	3.82/1.25	b	a	3.95/1.25 4.01 ± 0.86	b	a	4.80/1.40	b	b	5.23/1.86	b	b
	12	3.84/1.00	b	a	3.93/0.92 3.99 ± 0.82	b	a	4.48/1.44	b	b	5.01/2.20	b	b
	%	−20.71/23.52		a	−14.90/22.47		a	−15.29/20.70		a	−17.32/22.80		a
ARA/EPA	3	18.64/5.24	a	a	18.04/7.95	a	a	20.48/15.00	a	a	35.68/29.04	a	b
	6	18.13/12.13	a	a	19.63/13.42	a	a	22.58/12.25	a	a,b	30.30/21.87	a	b
	9	15.48/8.91	b	a	15.86/10.38	b	a	18.61/12.46	b	a,b	24.28/17.55	b	b
	12	14.49/13.03	b	a	14.88/10.56	b	a	18.65/11.39	b	a	23.44/17.56	b	b
	%	−17.29/29.16		a	−19.36/54.76		a	−19.60/44.12		a	−26.90/43.86		a
ARA/DHA	3	3.85/1.88	a,b	a	4.01/1.80	a	a	4.73/1.75	a	b	6.05/2.36 6.12 ± 1.78	a	c
	6	3.72/1.39	a	a	3.42/1.40	a	a	5.04/2.24	a	b	6.08/2.84 6.24 ± 2.08	a	b
	9	3.40/1.11	b	a	3.48/1.27	b	a	4.47/1.61	b	b	5.52/2.79 5.33 ± 1.85	b	b
	12	3.35/1.14	b	a	3.58/1.20	b	a	4.32/1.64	b	b	5.49/2.69 5.34 ± 1.92 1.92	b*	b
	%	−10.36/22.12		a	−11.34/20.52		a	−12.69/22.14		a	−12.93/24.30		a
LA/ALA	3	72.70/30.24 72.31 ± 20.14	a	a	64.80/29.33 1.10 ± 0.26	a	a	61.76/34.36 62.10 ± 22.81	a	a	63.42 ± 27.07	a	a
	6	47.57/28.60	b	a	53.57/34.28	a,b	a	46.94/23.12	a,b	a	48.80/39.49 55.93 ± 22.88	a,b	a
	9	47.67/27.11	b	a	44.51/33.87	b	a	50.17/26.44	a,b	a	47.47/28.10 48.45 ± 19.10	b	a
	12	49.09/28.03	b	a	47.79/30.76	a	a	45.63/34.42	b	a	49.47/26.40 51.30 ± 18.23	b	a
	%	−30.53/41.04		a	−8.70/57.71		a	−20.24/47.22		a	−21.72/47.63		a
ARA/LA	3	1.14/0.33 1.16 ± 0.19	a	a	1.08/0.33	a	a,b	1.04/0.30	a	b	0.90/0.21 0.91 ± 0.15	a	c
	6	1.25 ± 0.28	b	a	1.14/ 0.29 1.15 ± 0.19	a	b	0.97/0.29 1.03 ± 0.24	a	c	0.92 ± 0.17	a	d
	9	1.24 ± 0.18	b	a	1.11/0.26	a	b	1.04/0.34 1.05 ± 0.24	a	b	0.92 ± 0.16	a	c
	12	1.33 ± 0.25	c	a	1.10/0.22 1.15 ± 0.20	a	a	1.03/0.28 1.08 ± 0.21	a	a	0.96 ± 0.19	a	b
	%	16.03/20.65		a	6.04/22.88		a,b	1.15/28.25		b	6.53/18.75		a,b

Variable expressed as mean (±SD) and/or as median/IQR depending on the statistical test that was performed; ◊ comparison of time points within diet groups; Δ comparison between diet groups at individual time points; groups without a common letter are significantly different, *p* < 0.05; WD = omnivores, Flex = flexitarians, VG = vegetarians, VN = vegans, m = men, w = women, t = time point (month), % = percentual change from start of intervention, n = omega, ARA = arachidonic acid, EPA = eicosapentaenoic acid, DHA = docosahexaenoic acid, LA = linoleic acid, ALA = α-linolenic acid.

At follow-up the *n-*6/*n-*3 ratio remained stable, but vegetarians and vegans had higher ratios compared to omnivores (*p* < 0.02). Due to the ALA decrease, the LA/ALA ratio increased in all groups (*p* = 0.002). ARA/EPA ratios also increased, while ARA/DHA ratios decreased in several groups (*p* ≤ 0.01), with vegans showing a higher ARA/DHA ratio overall (*p* = 0.02). ARA/LA ratios increased in flexitarians at follow up (*p* = 0.001) and distinct group differences emerged with lower values by increased exclusion of animal products (*p* ≤ 0.02) (Supplementary Table S2).

Calculations for D9D remained unchanged across all groups over the study period. C16:1*n-*9/16:0 and C18:1*n-*9/C18:0 were similar between groups at baseline. After the intervention, C18:1*n-*9/C18:0 was higher in flexitarians, vegetarians, and vegans compared to the Western diet group (*p* ≤ 0.03), while vegans had lower ratios of C16:1*n-*9/16:0 (*p* < 0.001). The D5D also showed no within-group changes over time. However, *n*-6 D5D (C20:4*n-*6/C20:3*n-*6) decreased in vegetarians at month 12 compared to the Western diet group (*p* ≤ 0.03), with lower values in vegans compared to omnivorous groups (*p* ≤ 0.02). Lower levels of *n*-3 D5D (C20:5*n-*3/C20:4*n-*3) were found in vegetarians and vegans initially (*p* ≤ 0.02) which were no longer significantly different at the end. *n*-6 D6D (C20:3*n-*6/C20:2*n-*6) remained stable over time, with flexitarians and vegans showing lower levels than the Western diet group at the end (*p* = 0.03). Another calculation of *n*-6 D6D (18:3*n-*6/C18:2*n-*6) showed a significant increase in the omnivorous groups about 12.3–22.8% during the intervention (*p* < 0.001), with no significant changes from baseline across all groups. At the start, vegetarians had higher values than flexitarians and vegans (*p* = 0.01), but no differences were observed at the end. In addition, *n*-3 D6D (C20:5*n-*3/C18:3*n-*3) increased in vegans about 16.9% during the intervention (*p* = 0.04), with no significant difference in this incline from other groups. Yet, vegans consistently had the lowest absolute amounts (*p* < 0.001), while Western diet subjects had the highest levels of all groups at both baseline and the end (*p* ≤ 0.02). At month 12, the ratio differed across all groups with increasing exclusion of animal products (WD > Flex > VG > VN, *p* < 0.05; Supplementary Table S3).

The calculated *n*-6 D5D and D6D activity (C20:3*n*-6/C18:2*n*-6) decreased in all groups at follow-up (*p* < 0.001), whereas the *n*-3 D5D activity increased in Western diet subjects and vegans (*p* < 0.001). In addition, vegans exhibited an augmentation of *n*-3 D6D activity (C20:5*n*-3/C18:3*n*-3) in the follow-up assessment (*p* = 0.01). Concerning group differences, *n*-6 D5D was still lower in vegetarians and vegans compared to the omnivorous groups (*p* ≤ 0.03), whereas the group differences in *n*-3 D6D (C20:5*n*-3/C18:3*n*-3) were equal compared to the end of the intervention (*p* < 0.05) (Supplementary Table S4).

### 3.3 Subgroup analysis of potential influencing factors of ALA-conversion

**Figure 3:**
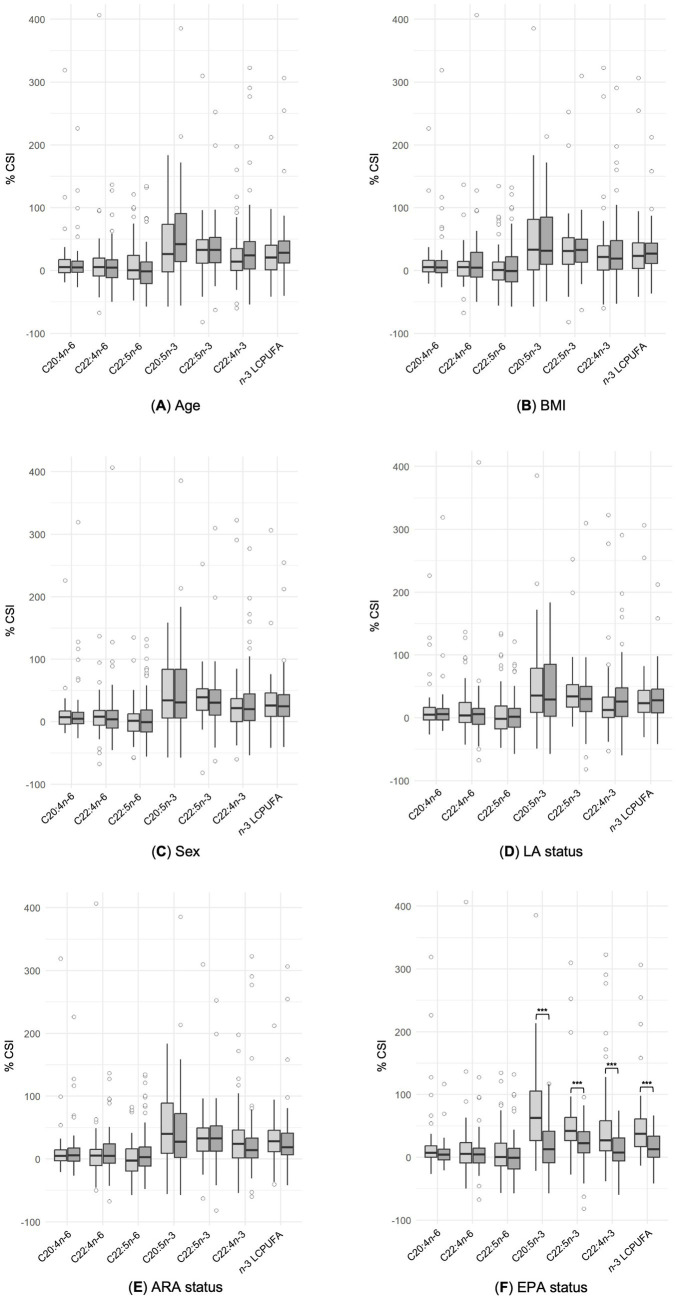
Comparison of % change from start of Intervention (%CSI) of selected *n*-6 and *n*-3 PUFA fatty acids according to age **(A)**, BMI **(B)** sex **(C)**, LA status **(D)** ARA status **(E)** and EPA status at the start of the intervention **(F)**. The results are shown as box plot with median (indicated as lines). Light grey box plots show the subgroup with a low starting value, whereas dark grey box plots indicate a higher starting value. Regarding sex, data of men are presented in light gray, while data of women are represented by dark grey box plots. [Mann–Whitney *U* Test, (***p < 0.001)].

To evaluate the conversion rate of ALA into its LC metabolites, we examined sex (men vs. women), BMI (<22.42 vs. ≥22.42), age (<28 vs. ≥28), LA status (<12.59 vs. ≥12.59% FAME), ARA status (<13.29 vs. ≥13.29% FAME) and EPA status (<0.58 vs. ≥0.58% FAME) at the start of the intervention as potential influencing factors. The subgroups mentioned above were firstly formed within the four dietary patterns (Supplementary Table S5). In accordance with the comparison of %CSI in erythrocyte lipids no group differences were observed between participants with high or low baseline levels across the dietary patterns (Supplementary Table S5). Because of this, and to increase the sample size, the division into diet groups was removed. When disregarding dietary patterns, no difference emerged for %CSI of ARA, EPA, DPA, DHA or *n*-3 LCPUFA regarding sex, BMI and age. Moreover, categorization by LA and ARA status showed no significant differences in the %CSI of selected fatty acids between subjects with high or low values at the start of the intervention (Figures 3A-E). However, participants with low baseline EPA status showed a higher %CSI in EPA (62.9% vs. 13.0%, *p* < 0.001), DPA (41.9% vs. 22.3%, *p* < 0.001), and DHA (27.0% vs. 7.6%, *p* < 0.001) levels compared to those with a higher status at the beginning (*p* < 0.001; Figure 3F). Using Chi^2^-test, a significant connection was found between the dietary pattern and the EPA baseline value [χ^2^(3) = 10.54, *p* = 0.015], with vegans showing a higher proportion of subjects with a low EPA baseline value (71.4%).

To investigate the impact of micronutrient status on conversion of ALA into LC metabolites a correlation analysis between micronutrient status (selenium, zinc, copper, calcium, potassium, ferritin, transferrin, folic acid, biotin, holo-transcobalamin (holo-TC), vitamins A, B_1_, B_2_, B_6_, B_12_, C, D, E) at the start of the intervention and the %CSI of ARA, EPA, DPA, DHA and LC *n*-3 was conducted on the whole study collective. Serum selenium concentration showed a small correlation with the %CSI of ARA (*r* = 0.173, *p* = 0.03). Furthermore transferrin correlated slightly with the %CSI of EPA (*r* = −0.190, *p* = 0.02), while serum iron and transferrin saturation showed minor correlation with the %CSI of ARA (*r* = 0.192, *p* = 0.02; *r* = 0.181, *p* = 0.02), EPA (*r* = 0.189, *p* = 0.02; *r* = 0.217, *p* < 0.001), DPA (*r* = 0.247, *p* < 0.001; *r* = 0.254, *p* < 0.001) DHA (*r* = 0.238, *p* < 0.01; *r* = 0.220, *p* < 0.01) and *n*-3 LCPUFA (*r* = 0.263, *p* < 0.001; *r* = 0.259, *p* < 0.001). No significant correlation emerged for ferritin or any other micronutrient and vitamin with the respective fatty acids or their %CSI (Supplementary Figure S1).

## 4 Discussion

The NuEva study aimed to establish nutritional concepts to ensure adequate nutrient supply and reduce cardiovascular risk factors with individual, practicable dietary recommendations for different dietary patterns (Western diet, flexitarian, vegetarian and vegan). The aim was to collect valuable information for planning a healthy and nutritionally adequate diet for the four dietary patterns, as there is a lack of reliable data, especially for vegans. The intervention included seasonal, energy-adapted and nutrient-optimized menu plans, in which the fatty acid quality was optimized. One main focus was the improvement of *n*-3 LC PUFA status in the variety of dietary patterns and the evaluation of the impact of influencing factors on conversion of plant-based ALA into its LC metabolites. As vegetarians and vegans omit marine food sources like fish, achieving the recommended intake of *n*-3 LCPUFA is challenging. Besides consumption of seaweeds and microalgae, they rely on the conversion of ALA to EPA, DPA and DHA (29). However, the intake (< 250 mg/d) and status (*n*-3 Index 4–6%) of these *n*-3 LCPUFA of people in central Europe (including Germany) is also too low in the context of preventing non-communicable diseases (18, 30, 31). Therefore, flaxseed oil was regularly included in the NuEva menu plans, whereby an average ALA quantity of at least 3 g/d (∅ 4.6–5.8 g/d) was achieved. Simultaneously, the LA/ALA ratio in the diet was reduced to < 5:1 (∅ 2.2–2.7:1). A low LA/ALA ratio is associated with an increased conversion of ALA into *n*-3 LCPUFA in erythrocyte membranes (32), resulting in an effective way of improving *n*-3 PUFA status (22).

Despite the adaption of LA/ALA ratio, vegans had the highest LA concentration in erythrocyte lipids over the entire period compared to the other dietary patterns, which is mainly due to the higher LA content in plant foods (33–35). Comparable studies described also higher LA concentrations in erythrocyte lipids of vegans and vegetarians (36, 37). However, after the intervention period ARA concentrations were lower in vegetarians and vegans compared to omnivores. As vegetarians and vegans consume little to no ARA through the diet, ARA results mainly from endogenous synthesis of LA and dihomo-*γ*-linolenic. In the Western diet group, ARA levels increased significantly by 12% towards the end of the study, which is likely due to an increase in D6D activity from the intake of flaxseed oil. Although the intake of meat and meat products was not increased in the Western diet group through the intervention, these foods are known to be an important source of ARA (38) and are associated with increased levels of this fatty acid (39), so daily consumption of these products may also have contributed to the observed increase.

Since ALA makes up the largest proportion of fatty acids in flaxseed oil (40), the observed increase in ALA concentration was expected and confirms the compliance of flaxseed oil intake of the subjects. This increase was evident for both plasma and erythrocytes and reached a steady state at month 6 in all groups. This is consistent with the fact that an increase in ALA concentration causes catabolism or increased storage of this fatty acid (20). In principle, three metabolic pathways of ALA are possible after dietary intake. Incorporation into adipose tissue for storage, *β*-oxidation for energy production and conversion into *n*-3 LCPUFA (41). While both plasma and erythrocyte *n*-3 LCPUFA levels increased over the course of the study, erythrocyte lipids provided a more accurate and consistent reflection of the effects of increased ALA intake on EPA, DPA, and DHA status. As plasma lipids primarily reflect short-term fatty acid intake over the last previous days, we decided to focus on erythrocyte lipids, which provide a more reliable marker of the continuous incorporation of fatty acids into erythrocyte membranes due to the gradual turnover of erythrocytes (~120 days), also corresponding to the time interval between study visits. Overall, erythrocyte lipids were shown to be a more stable model for assessing the impact of ALA supplementation on *n*-3 LCPUFA status. Here a substantial proportion of ALA was converted to EPA and achieved an increase of up to 30% and in vegans even up to 41% in erythrocyte lipids. In addition, the conversion of ALA to DPA was reflected in a similar increase of this fatty acid of up to 41%. The %CSI of both fatty acids did not differ significantly between the four dietary patterns. In addition, levels of DPA were comparable between the groups at all time points, suggesting a reservoir function for conversion to DHA or retroconversion to EPA. Differences in EPA and DHA content, but comparable DPA content between omnivores and vegans, have so far only been described in a cross-sectional study (42).

To date only a few studies with small numbers of participants have investigated interventions with flaxseed oil on vegans and vegetarians and did not compare the results to omnivores groups. In contrast to our data, Fokkema et al. (2000) observed no changes in erythrocyte *n*-3 LCPUFA in 12 vegans supplemented with 2 g ALA/d for 4 weeks (43). However, Li et al. (1999) reported an EPA increase of 50 and 150% after 4 weeks in erythrocyte lipids of 22 vegetarians consuming 3.7 or 15.4 g ALA/d respectively, indicating a dose-dependent response. While this result was in line with our finding, the conversion to DPA was only evident for the group with higher ALA intake (47%) (44). Studies, that did not distinguish between different dietary patterns using higher ALA dosages of 13–16 g/d for 12 weeks reported a remarkable increase in ALA (296–385%), EPA (37–112%) and DPA (11–39%) in erythrocyte lipids (45, 46). In addition, studies with moderate ALA intakes also achieved consistent increases in both EPA and DPA. Kuhnt et al. (2016) used flaxseed oil supplying 5 g of ALA/d for 8 weeks resulting in a more pronounced ALA increase (201%), a comparable rise in EPA (32%), but a lower increase in DPA (12%) in erythrocytes in omnivores (47). Barceló-Coblijn et al. (2008) used ALA doses closely aligned with the NuEva study (2.4 g and 3.6 g/d) through flaxseed oil capsules over 6 weeks (48). They observed a dose-depended increase in ALA (26–67%) and EPA (32–65%) levels, while a significant rise in DPA (29%) was only observed with an ALA intake of 2.4 g/d (48). These results support our finding that flaxseed oil supplying moderate amounts of ALA can enhance conversion and lead to measurable increases in EPA and DPA.

Interestingly, we also observed an increase in DHA between 13 and 26%. While the conversion of ALA to EPA and DHA is seen less critically in the literature, the efficacy of ALA conversion to DHA is still under debate (49, 50). In accordance most studies mentioned above found no increase in DHA (43, 44, 48) or even reported a decrease through the flaxseed oil/higher ALA intake (45–47). One reason could be the partly higher amounts of ALA compared to NuEva, which could have favored increased *β*-oxidation, as ALA belongs to the PUFA with the highest oxidation rate and thus possibly less ALA was available for conversion (45, 46). In addition, mostly shorter periods (6–12 weeks) were investigated. Our data underscore the need for longer supplementation periods with moderate dosage, as we also observed an increase in DHA after the 9-month intervention with moderate doses of flaxseed oil. A Japanese cohort on elderly subjects investigated a moderate but long-term increase of dietary ALA (3 g/d) in combination with an *n*-6/*n*-3 ratio of 1:1 through the diet (51). After 3 months, no effects were observed in DHA, but after 10 months, serum DHA increased by approximately 21% (51). This is in accordance with our data, suggesting that the duration of ALA administration and the background diet may be decisive in increasing DHA status.

In the next step, we aimed to examine the influence of various factors on the conversion of ALA into its LC metabolites. D6D and D5D regulate the desaturation of LA and ALA to their respective LC PUFA metabolites. The determination of product/precursor ratios is a widely used method in epidemiological studies to estimate D6D and D5D activity, as studies measuring mRNA expression or protein levels of desaturases is difficult in humans, since the most relevant tissue to study is the liver (52). Typically, *n*-6 fatty acids are used for this estimation, as the intake of *n*-6 fatty acids are higher in Western diets. Examination of estimated desaturase activities (high D6D, low D5D) have shown to predict metabolic risks like type 2 diabetes mellitus (53), metabolic syndrome and total as well as cardiometabolic mortality (54, 55). Here, an increase in D6D (C20:3*n*-6/C18:2*n*-6) was observed in the Western diet group, which was significantly different from the vegetarians and vegans by the end of the intervention, while D5D did not change in any group. In comparison, short-term dietary intervention with plant oils rich in ALA (rapeseed oil, 6.6 g ALA/d or flaxseed oil 12.9 g ALA/d) have resulted in a decrease in the D6D (45, 52) and an increase in D5D activity (52). However, estimated desaturase activities in interventions should be interpreted with caution. Product/precursor ratios can be influenced by several factors, particularly the dietary intake of the involved fatty acids. In addition, the observed changes may result not only from enzymatic modulation but also from substrate competition and feedback mechanisms, which cannot be accurately reflected in the indices (56).

To specify the factors that might have influenced the conversion into LC PUFA, we therefore conducted a subgroup analysis considering the %CSI of relevant fatty acids. The comparison of the %CSI of the *n*-3 LCPUFA shows no difference in conversion rate in dependence of the specific dietary pattern. Therefore, we formed the dietary subgroups in a whole collective for further analysis of influencing factors.

Contrary to many previous publications, we found no sex-specific differences in the conversion of ALA into the LC metabolites. The literature often reports that women, possibly due to estrogen-related increases, utilize more ALA for conversion rather than *β*-oxidation, thus converting ALA more efficiently into DHA (21, 57). A systematic review based on 51 publications also revealed higher plasma DHA (difference 0.13 %FAME) values in women aged 13–15 years compared to men (58). However, in the NuEva cohort, no differences in the percentual change of EPA, DPA or DHA were observed between women and men. Possible reasons include the unbalanced gender ratio, with a significantly higher proportion of women (73%).

We also did not observe differences between the %CSI of *n*-3 LCPUFA dividing in higher and lower BMI (<22.42 vs. ≥22.42), or age (<28 vs. ≥28). Most interventions studying the effect of flaxseed oil on EPA, DPA and DHA do not differentiate between subjects with different age or BMI. A systematic review evaluating the association between non-dietary factors and *n*-3 LCPUFA found no clear results for both parameters. For BMI negative or no associations with erythrocyte *n*-3 LCPUFA were reported (59). Studies that found a negative effect were mainly subjects with higher BMI (>25 kg/m^2^) compared to NuEva, where the cut off value for subgroup analysis was 21 kg/m^2^. The authors suggest that in overweight or obese individuals it is possible that a higher susceptibility to peroxidation occurs (59). It is therefore likely that there is no significant impact on conversion in individuals with a normal BMI range. In addition, erythrocyte EPA and DHA tended to be positively associated with age in adults (approximately 19% increase from age 20 to 75 years) (59), which we did not observe for the subjects older than 28 years. In accordance, Schiller et al. (2016) found only weak associations between age and estimated desaturase activity in the EPIC Potsdam study, suggesting that this factor may have less direct impact on enzyme regulation (60).

D6D and D5D are encoded by the *FADS2* and *FADS1* genes, respectively. Numerous single nucleotide polymorphisms have been described for these genes, which modify the activity of desaturation and the lipid composition (61). In the EPIC Potsdam cohort, it was shown that single nucleotide polymorphisms can explain up to 24% of the variance in *n*-6 and *n*-3 LC PUFA (62). Ameur et al. (2012) performed genome-wide genotyping in 5,652 individuals from five European populations and observed two common FADS haplotypes (63). The more common haplotype D predicted more active conversion than the less common haplotype A, with homozygous subjects for haplotype D showing 24% higher DHA and 43% higher ARA levels (63). In addition, a randomized controlled trial was conducted to investigate the effect of a diet enriched with flaxseed oil (20.6 g ALA/d), compared with a Western diet (1.3 g ALA/d) as well as *FADS1* and *FADS2* single nucleotide polymorphisms on plasma fatty acids and [U-^13^C]ALA metabolism. The authors demonstrated that minor allele variants in *FADS1* and *FADS2* were associated with lower desaturase activity, as evidenced by reduced plasma [^13^C]EPA and the percentage composition of EPA, irrespective of the dietary treatment group (64). It can therefore be assumed that genetic make-up had a relevant influence on the efficiency of the conversion in our study, even though we have not been able to quantify this aspect in the absence of genotype data.

Besides biological variables, we also wanted to investigate the effect of nutrient status on ALA conversion. Regarding micronutrients, the physiological connection between zinc, iron, vitamin A, folate, vitamin B_12_ and ALA conversion has mainly been investigated in mouse models and was described in the review by Gonzales et al. (65). In the NuEva study, the menu plans optimized the intake of micronutrients based on the D-A-CH reference values. Comprehensive nutrient analyses were carried out to assess the impact of the meal plans on nutrient status. However, only small or no correlation was found for micronutrients and the change of LC PUFA. Consequently, it is likely that other factors, may play a more significant role in influencing ALA conversion.

Due to the competition of *n*-3 and *n*-6 PUFA for the same enzymes systems and incorporation into cell membranes we investigated the status of LA and ARA as potential influencing factor of ALA conversion. When subjects were stratified into high and low LA (< 12.59 vs. ≥ 12.59% FAME) or ARA status (< 13.29 vs. ≥ 13.29% FAME) at the beginning of the intervention, we found no significant differences in the %CSI in *n*-3 LCPUFA. This suggests that initial *n*-6 PUFA status alone may not be a decisive factor in determining efficiency of ALA conversion under given intervention conditions. In addition, Drobner et al. (2023) showed, that the change in LA concentration throughout the study seems to have less of an impact on the ALA conversion as well, when a high intake of ALA through flaxseed oil was applied (46). Regarding *n*-3 status, our subgroup analysis revealed that a lower EPA status (< 0.58% FAME) at the beginning of the intervention was a key determinant of efficient ALA conversion into EPA, DPA and DHA. This observation is consistent with the previous finding of Drobner et al., where individuals with lower EPA status at baseline (< 0.9% FAME) showed a stronger increase in EPA levels (79% vs. 29%) compared to those with higher baseline levels (46). These results support the broader concept that a low *n*-3 LCPUFA status is associated with an improved conversion rate from ALA, likely due to reduced feedback inhibition along the desaturation pathway. Even if the dietary pattern did not influence the conversion, it is notable that vegans made up most subjects with low EPA concentrations (71%). This is in line with the fact that a greater theoretical potential for improving their *n*-3 PUFA status through endogenous conversion from ALA is suggested for vegetarians and vegans (66). In addition, it was already described, that additional ALA intake does not necessarily achieve a measurable effect in individuals with adequate or high baseline *n*-3 LCPUFA levels (67). Overall, our data suggest that the initial status of *n*-3 LCPUFA, especially EPA, has the greatest influence on the efficiency of ALA conversion when moderate amounts of flaxseed oil are consumed.

The NuEva study has several strengths but also some limitations. The design of the NuEva study belongs to the strengths as the comprehensive nutritional approach over a period of more than nine months has not yet been investigated, particularly in dietary pattern which avoid n-3 LCPUFA sources of marine origin such as vegetarians and vegans. The intervention based on a variety of prepared menu plans ensuring an optimized intake of energy, macro- and micronutrients with particular emphasis on ensuring a ratio of LA/ALA < 5:1.

During the nine-month intervention, blood samples were taken every three months to examine changes in parameters over time. To increase compliance, participants were able to choose from an extensive repertoire of 80 daily menus tailored to the different seasons and their dietary preferences. Regular consultations were held on relevant nutritional aspects and possible challenges in implementing the menu plans, along with the provision of study foods such as flaxseed oil.

The effects of implementing the provided meal plans on nutritional status, with a focus on fatty acid status, were assessed regularly. However, the implementation of the meal plans in everyday life depended heavily on the motivation of the participants. Compliance was measured using self-reported information in combination with an analysis of nutritional status, which is influenced by various factors.

A major limitation is the lack of a control group following their habitual diet without implementation of the holistic nutritional concepts in dependence of the dietary habits. Furthermore, we decided not to randomize our participants who were required to follow one of the four diets under investigation for at least one year prior to the start of the NuEva study in order to identify potential critical nutrients resulting from long-term adherence. The absence of randomization due to the predefined inclusion criteria, introduces risk of selection bias.

In addition, the gender distribution was unbalanced, with a predominance of female participants, this may compromise generalizability.

As a further strength, we investigated the effect of a moderate dose of ALA, which can be easily integrated into everyday life using the recipes provided (no isolated dietary supplement). In this scenario, we were able to show that moderate long-term intake of ALA is suitable for improving EPA and DHA concentrations, especially in participants with low baseline status. As a limitation in this context, based on our data in healthy participants, it remains difficult to estimate the effects of improvements in n-3 LCPUFA status on health outcomes such as inflammatory processes, cognitive function, or glucose and lipid metabolism, which can be modulated by long-term n-3 LCPUFA supplementation or regular intake through diet.

To strengthen causal inference, future studies should adopt randomized, placebo-controlled, parallel-group designs with multiple ALA doses to establish dose–response curves and minimize bias. In this context, studies providing standardized diets systematically varying in their ALA and LA intake will provide further data on its competitive impact on metabolism of LCPUFA. In addition, stable-isotope tracer methodologies can be used to directly quantify fractional conversion rates to EPA, DPA, and DHA. Fatty Acid Desaturase (FADS) 1/FADS 2 genotyping and gene expression analyses could be included to identify genetic modifiers of metabolic efficiency.

In addition, the influence of other macro- and micronutrients on the conversion of ALA into its LC metabolites could be further investigated, although no influencing factors could be identified in this regard in the NuEva study.

## 5 Conclusion

The NuEva study demonstrates that systematic long-term dietary intake of ALA, accompanied by a controlled background diet, leads to a significant increase in EPA, DPA and DHA concentrations in plasma and erythrocyte lipids, independent of the dietary pattern. However, the changes observed in erythrocyte lipids were more pronounced and consistent, highlighting erythrocytes as a suitable model to reflect the effects of ALA supplementation on *n*-3 LCPUFA at 3-monthly intervals. While sex, BMI, age, LA status and ARA status did not affect the conversion of ALA to *n*-3 LCPUFA, the increase of EPA, DPA and DHA was stronger in subjects with lower EPA levels at the beginning of the intervention. Despite the significant improvements from the intervention with flaxseed oil, the vegans and vegetarians still have lower *n*-3 LCPUFA concentrations than omnivores, but on average surpass omnivorous levels at the beginning of the intervention. In this context, it should be noted that ARA concentrations are also lowest in vegans and vegetarians. To counteract the absence of dietary EPA, DPA, and DHA typically obtained from fish, vegetarians and vegans are advised to regularly consume walnuts, flax-, chia- and hempseeds as source of the precursor ALA in combination with EPA- or DHA-rich microalgae oil to improve their long-term supply of *n*-3 LCPUFA.

## Data Availability

The raw data supporting the conclusions of this article will be made available by the authors, without undue reservation.
