# Supplementary material for: Impact of α-linolenic acid supplementation on long-chain n-3 fatty acid profiles in Western, flexitarian, vegetarian, and vegan diets
Source: Front Nutr. 2025 Dec 15;12:1727308. doi: 10.3389/fnut.2025.1727308 (PMC12745237; doi:10.3389/fnut.2025.1727308)
Supplement: Supplementary file 1 [file Data_Sheet_1.pdf]

Supplementary material

Table S1: Saturated, monounsaturated and trans fatty acid profile (% FAME) in erythrocyte lipids during the intervention according to diet group

	t	**WD**<br>(27 w, 11 m)	◊	Δ	**Flex**<br>(37 w, 8 m)	◊	Δ	**VG**<br>(30 w, 13 m)	◊	Δ	**VN**<br>(28 w, 14 m)	◊	Δ
**SFA**													
C14:0	3	0.25 / 0.06	a	a,b	0.21 / 0.10<br>0.22 ± 0.06	a	a,c	0.27 / 0.09	a	b	0.18 / 0.09	a	c
	6	0.23 / 0.09	a	a	0.23 / 0.07<br>0.24 ± 0.07	a	a	0.23 / 0.12	a	a	0.14 / 0.08	a,c	b
	9	0.31 / 0.10	b	a	0.28 / 0.11<br>0.29 ± 0.08	b	a	0.33 / 0.11	b	a	0.22 / 0.12	b	b
	12	0.17 / 0.08	c	a	0.23/ 0.09<br>0.24 ± 0.07	a	b	0.24 / 0.10	a	b	0.15/ 0.09	b,c	a
	%	-32.97 / 35.39		a	-3.78 / 63.25		b	-21.71 / 50.66		a	-19.45 / 45.46		a
C15:0	3	0.15 / 0.03	a	a	0.15 / 0.04<br>0.15 ± 0.04	a,b	a	0.17 / 0.06	a	a	0.08 / 0.03	a	b
	6	0.14 / 0.03<br>0.14 ± 0.03	b	a	0.15 ± 0.03	a,b	a	0.15 / 0.05<br>0.15 ± 0.04	b	a	0.08 / 0.04<br>0.08 ± 0.02	b	b
	9	0.16 / 0.05	a	a	0.16 / 0.03<br>0.16 ± 0.03	a	a	0.17 / 0.04	a	a	0.08 / 0.03	a	b
	12	0.12 / 0.04	c	a	0.15 / 0.05<br>0.14 ± 0.03	b	b	0.13 / 0.03	c	b	0.07 / 0.03	c	c
	%	-24.92 / 17.91		a	-5.77 / 35.69		b	-20.81 / 27.35		a	-26.67 / 27.53		a
C16:0	3	23.29 / 2.95	a	a	22.65 / 1.59	a	a	23.33 / 3.43	a	a	22.96 / 2.41<br>22.94 ± 1.94	a	a
	6	22.52 / 3.33<br>22.25 ± 2.36	a	a	22.81 / 2.63<br>22.69 ± 2.30	a	a	22.35 / 5.23<br>22.66 ± 3.71	a	a	21.55 / 2.86<br>21.55 ± 2.59	b	a
	9	21.84 / 1.94	a	a	22.00 / 1.84	b	a	22.43 / 1.96	a	a	21.44 / 1.70<br>21.64 ± 1.43	b	a
	12	18.00 / 3.25	b	a	20.80 / 3.18	b	b	21.01 / 3.09	b	b	18.83 / 3.45<br>19.27 ± 2.31	c	a
	%	-20.67 / 20.28		a	-8.87 / 16.47		b	-14.14 / 24.74		a,b	-15.96 / 17.76		a,b
C17:0	3	0.29/ 0.04<br>0.28 ± 0.03	a	a	0.28 / 0.08	a	a	0.29 / 0.07	a	a	0.23 / 0.06	a	b
	6	0.25 / 0.06<br>0.25 ± 0.04	b	a	0.27 / 0.06	b	a	0.26 / 0.05	b	a	0.21 / 0.07	b	b
	9	0.25 / 0.05<br>0.25 / 0.03	b	a	0.27 / 0.05	b,c	a	0.26 / 0.06	b	a	0.20 / 0.06	b	b
	12	0.23 ± 0.03	c	a	0.26 / 0.05<br>0.25 ± 0.04	c	b	0.24 / 0.05<br>0.24 ± 0.03	c	a,b	0.20 / 0.05<br>0.20 ± 0.03	c	c
	%	-15.76 / 10.95		a	-13.73 / 14.20		a	-15.13 / 7.25		a	-18.01 / 9.75		a
C18:0	3	12.52 / 2.74	a	a	12.24 / 2.20	a	a	12.46 / 2.70	a	a	12.49 / 1.59	a	a
	6	13.11 / 1.96	a	a,b	12.53 / 2.22	a	a	13.00 / 3.21	a	a,b	13.42 / 2.22	b	b
	9	12.27 / 1.29<br>12.33 ± 1.05	a	a*	12.36 / 1.59<br>12.20 ± 1.18	a	a*	12.43 / 1.53	a	a	12.63 / 1.88<br>12.82 ± 1.37	a,b	a
	12	13.01 / 1.00	a	a	12.05 / 1.59	a	b	12.53 / 1.49	a	a,b	12.93 / 1.30	a,b	a
	%	6.34 / 23.34		a	-2.64 / 22.91		a	-2.81 / 22.39		a	3.82 / 19.02		a
C20:0	3	0.07 / 0.02	a	a	0.08 / 0.03	a	a	0.08 / 0.02	a	a	0.10 / 0.02	a	b
	6	0.09 / 0.01	c	a	0.10 / 0.03	b	a,b	0.10 / 0.03	b	b	0.13 / 0.03	c	c
	9	0.12 / 0.04	b	a	0.11 / 0.03	c	a	0.12 / 0.04	c	a	0.14 / 0.04	b	b
	12	0.10 / 0.04	b,c	a	0.11 / 0.04	c	a	0.13 / 0.05	c	a	0.14 / 0.04	b,c	b
	%	27.78 / 44.65		a	38.36 / 53.09		a	54.32 / 68.71		a	37.68 / 48.24		a
Σ SFA	3	38.64 / 1.89	a	a	38.36 / 2.31	a	a	39.13 / 2.75	a	a	38.36 / 2.29	a	a

	6	41.33 / 2.11	b	a	40.77 / 2.27	b	a,b	40.20 / 3.98	b	a,b	39.87 / 2.50	b	b
	9	40.60 / 1.60	b	a	40.12 / 2.26	a,b	a,b	40.29 / 1.88	b	a	39.55 / 1.86	b	b
	12	36.64 / 4.02	a	a	39.19 / 3.87	a	b	38.97 / 2.89	a	b	37.54 / 3.40	a	a
	%	-4.21 / 12.26		a	0.62 / 13.08		a	-1.30 / 12.27		a	-4.07 / 10.77		a
**MUFA**													
C16:1*n*-7	3	0.24 / 0.09	a	a	0.24 / 0.14	a	a	0.27 / 0.18	a	a	0.18 / 0.09	a	b
	6	0.23 / 0.09	a,b	a	0.22 / 0.07	a	a	0.20 / 0.09	b,c	a	0.13 / 0.04	b,c	b
	9	0.23 / 0.09	a	a	0.21 / 0.11	a	a	0.25 / 0.11	a,c	a	0.13 / 0.08	b	b
	12	0.21 / 0.07	b	a	0.21 / 0.10	a	a	0.21 / 0.08	b	a	0.11 / 0.08	c	b
	%	-19.72 / 35.65		a	-13.57 / 62.03		a	-27.46 / 45.95		a	-29.01 / 37.77		a
C18:1*n*-9	3	15.19 / 1.46	a,b	a	15.71 / 1.89	a	a,b	15.14 / 1.42	a	a	16.33 / 1.74	a	b
	6	15.53 / 1.49	a	a	15.29 / 1.42	a	a	15.48 / 1.06	b	a	16.78 / 1.85	b	b
	9	14.98 / 1.23<br>14.98 ± 1.04	a,b	a	14.75 / 1.33<br>14.98 ± 1.22	b	a	15.02 / 1.39<br>15.03 ± 1.18	a	a	15.94 / 1.65	a	b
	12	14.79 / 1.06	b	a	15.22 / 1.41	b	a	15.27 / 1.35	a,b	a	16.12 / 1.71	a	b
	%	-3.38 / 12.92		a	-2.42 / 12.18		a	1.36 / 8.79		a	-0.28 / 8.75		a
Σ MUFA	3	17.06 / 1.55	a	a	17.65 / 2.07	a	a,b	17.02 / 1.88	a	a	18.20 / 1.84	a	b
	6	17.39 / 1.66	a	a	17.06 / 1.48	a	a	17.32 / 1.16	b	a	18.53 / 1.89	b	b
	9	16.75 / 1.44<br>16.76 ± 1.08	a,b	a	16.63 / 1.46<br>16.73 ± 1.28	b	a	16.56 / 1.31<br>16.69 ± 1.27	a	a	17.70 / 1.95<br>17.69 ±1.09	b,c	b
	12	16.54 / 1.44	b	a	16.90 / 1.55	b	a	16.85 / 1.42	a	a	17.74 / 1.80	a,c	b
	%	-3.45 / 12.45		a	-3.21 / 12.40		a	-0.75 / 6.32		a	-1.96 / 7.56		a
**TFA**													
Σ TFA	3	0.37 / 0.07	a	a	0.36 / 0.09	a	a	0.39 / 0.13	a	a	0.27 / 0.09	a	b
	6	0.30 / 0.15	a	a	0.35 / 0.13	a	a,b	0.38 / 0.20	a	b	0.20 / 0.23	b	c
	9	0.34 / 0.14	a	a	0.36 / 0.12	a	a	0.40 / 0.14	a	b	0.22 / 0.10	b	b
	12	0.34 / 0.15	a	a	0.33 / 0.11	a	a	0.34 / 0.17	a	a	0.20 / 0.07	b	b
	%	-13.00 / 38.89		a	-7.75 / 37.75		a	-16.79 / 51.68		a,b	-25.99 / 32.33		b
CLA	3	0.10 / 0.03	a	a,b	0.08 / 0.05	a,b	a	0.10 / 0.04	a	b	0.02 / 0.02	a	c
	6	0.08 / 0.03	b	a	0.08 / 0.04	a	a	0.08 / 0.05	b	a	0.01 / 0.01	b	b
	9	0.09 / 0.04	a	a	0.09 / 0.02	b,c	a	0.10 / 0.04	a	a	0.02 / 0.02	a	b
	12	0.09 / 0.03	a	a	0.09 / 0.02	c	a	0.10 / 0.03	a	a	0.02 / 0.02	a	b
	%	-0.88 / 24.43		a,b	10.59 / 38.83		a	-10.92 / 28.58		b	-9.81 / 61.57		b

Variable expressed as mean (± SD) and/or as median / IQR depending on the statistical test that was performed; ◊ comparison of time points within diet groups; Δ comparison between diet groups at individual time points; groups without a common letter are significantly different, $p < 0.05$; Abbreviations: WD = omnivores, Flex = flexitarians, VG = vegetarians, VN = vegans, m = men, w = women, t = time point (month), % = percentual change from start of intervention, SFA = saturated fatty acids, MUFA = monounsaturated fatty acids, *n* = omega, TFA = trans fatty acids, CLA = conjugated linoleic acids

Table S2: Comparison of fatty acid profile (% FAME) in erythrocyte lipids from the end of the intervention (12 months) to follow-up (24 months)

	t	**WD**<br>(8 m, 19 f)	◊	Δ	**Flex**<br>(5 m, 30 f)	◊	Δ	**VG**<br>(11 m, 27 f)	◊	Δ	**VN**<br>(10 m, 16 f)	◊	Δ
**SFA**													
	12	0.17 / 0.08	<0.001	a	0.23 / 0.08<br>0.24 ± 0.06	<0.001	b	0.22 / 0.09<br>0.23 ± 0.07	<0.001	b	0.13 / 0.08<br>0.14 ± 0.05	<0.001	c
C14:0	24	0.32 / 0.10<br>0.31 ± 0.06		a	0.32 / 0.09<br>0.32 ± 0.07		a	0.36 ± 0.10		b	0.26 ± 0.09		c
	%	76.30 / 74.86		a	31.36 / 35.94		b	66.41 / 70.33		a	84.47 / 108.57		a
	12	0.12 / 0.04	<0.001	a	0.15 / 0.05	<0.001	b*	0.13 ± 0.03	<0.001	b	0.07 / 0.02	<0.01	c
C15:0	24	0.17 / 0.05		a	0.17 / 0.04		a	0.18 / 0.05<br>0.18 ± 0.04		a	0.09 / 0.04<br>0.09 ± 0.04		b
	%	48.03 / 60.27		a	20.45 / 27.03		b	41.17 / 35.49		a	37.12 / 52.84		a,b
	12	18.34 / 3.62	0.02	a	21.28 / 3.18<br>21.29 ± 2.41	<0.01	b	20.40 / 3.20<br>20.46 ± 1.89	n.s	b	18.75 / 3.16	n.s	a
C16:0	24	20.14 / 2.10		a	20.10 / 1.53<br>20.04 ± 1.02		a	20.52 / 1.58<br>20.83 ± 1.50		a	20.12 / 1.81		a
	%	13.06 / 19.36<br>9.49 ± 15.38		a	-4.88 / 10.36		b	2.59 ± 10.98		a	7.53 ± 17.31		a
	12	0.23 / 0.03	<0.001	a,c	0.25 ± 0.04	<0.001	a,b	0.24 ± 0.03	<0.001	b	0.20 ± 0.03	<0.001	c
C17:0	24	0.31 / 0.04		a	0.31 ± 0.04		a	0.32 ± 0.04		a	0.26 ± 0.05		b
	%	33.48 / 20.18<br>34.83 ± 15.10		a	25.99 / 19.63		a	31.75 / 18.07		a	28.92 / 17.64		a
	12	13.16 / 0.79	n.s	a	12.15 ± 1.30	<0.001	b	12.47 ± 1.03	n.s	b.c	13.04 ± 1.05	0.03	a,c
C18:0	24	13.79 /1.84		a	13.12 ± 1.19		a,b	12.57 ± 1.28		b	13.93 ± 1.44		a
	%	6.63 / 17.51		a	6.46 / 14.08		a	-0.41 / 12.98		a	6.40 ± 22.55		a
	12	0.10 / 0.04	<0.001	a	0.12 / 0.04	<0.001	a,b	0.12 / 0.05	0<.001	b	0.15 / 0.04	<0.001	c
C20:0	24	0.08 / 0.02		a	0.09 / 0.02		a,b	0.09 / 0.02		a,b	0.11 / 0.05		b
	%	-16.00 / 27.83		a	-22.82 / 0.89		a	-32.17 / 47.30		a	-27.14 / 43.38		a
	12	37.76 / 4.39	0.04	a,b	39.22 / 3.45<br>39.29 ± 2.50	n.s	a	38.81 / 2.63<br>38.58 ± 1.94	n.s	a	36.80 / 3.42	n.s	b
∑ SFA	24	39.04 / 1.37		a	38.68 / 1.51<br>38.52 ± 1.08		a,b	38.72 / 1.77<br>38.57 ± 1.53		a,b	37.88 / 1.69		b
	%	3.97 / 9.66<br>3.19 ± 6.85		a	-1.63 ± 6.30		b	0.23 ± 6.41		a,b	2.64 ± 9.61		a
**MUFA**													
	12	0.20 / 0.07	<0.001	a	0.20 / 0.12	<0.001	a	0.20 / 0.07<br>0.20 ± 0.05	<0.001	a	0.11 / 0.08	<0.001	b
C16:1*n*-7	24	0.27 / 0.09		a	0.25 / 0.12		a	0.28 / 0.12<br>0.27 ± 0.08		a	0.15 / 0.11		b
	%	39.67 / 45.22		a	16.55 / 38.36		a	28.16 / 36.15		a	18.83 / 48.64		a

	12	15.06 / 1.14	n.s	a	15.14 ± 1.28	<0.01	a	15.20 ± 1.18	<0.01	a	15.94 ± 1.16	n.s	b
C18:1*n*-9	24	14.80 / 1.05		a	14.74 ± 0.89		a	14.79 ± 1.05		a	15.57 ± 1.34		b
	%	-1.23 / 6.06		a	-2.66 / 7.89		a	-3.66 / 7.71		a	-1.14 / 7.20		a
	12	16.69 ± 1.25	n.s	a	16.97 / 1.92<br>16.85 ± 1.33	n.s	a	16.81 ± 1.23	n.s	a	17.67 ± 1.23	n.s	b
∑ MUFA	24	16.43 / 1.27<br>16.54 ± 1.12		a	16.62 / 1.38		a	16.53 / 1.80<br>16.53 ± 1.06		a	17.11 / 1.78<br>17.23 ± 1.38		a
	%	-1.18 / 6.57		a	-0.37 / 8.36		a	-2.06 / 7.69		a	-0.03 / 6.03		a
***n*-6 PUFA**													
	12	11.40 ± 1.46	0.03	a	12.18 ± 1.17	<0.01	b	12.96 ± 1.43	0.03	c	14.43 ± 1.53	<0.01	d
C18:2*n*-6	24	11.02 ± 1.30		a	11.67 ± 1.04		b	12.61 ± 0.96		c*	13.76 ± 1.74		d
	%	-3.49 / 6.17		a	-5.06 / 9.96		a	-2.76 / 8.65		a	-3.60 / 10.42		a
	12	0.04 / 0.02	n.s	a	0.04 / 0.02	n.s	a	0.04 / 0.02	n.s	a	0.04 / 0.02	n.s	a
C18:3*n*-6	24	0.04 / 0.02		a	0.04 / 0.02		a	0.04 / 0.02		a	0.04 / 0.02		a
	%	0.00 / 47.63		a	-10.00 / 56.04		a	1.19 / 49.00		a	-13.81 / 43.71		a
	12	0.20 / 0.03	n.s	a	0.23 / 0.04	<0.001	a,b	0.25 / 0.07	n.s	b	0.42 / 0.17	n.s	c
C20:2*n*-6	24	0.22 / 0.26		a	0.28 / 0.06		b.c	0.25 / 0.32		a,b	0.41 / 0.52		c
	%	11.79 / 132.26		a,b	25.40 / 18.25		a	16.24 / 129.44		a,b	11.29 / 123.06		b
	12	1.42 / 0.24<br>1.46 ± 0.25	n.s	a	1.38 /0.37<br>1.44 ± 0.32	<0.001	a	1.58 / 0.51<br>1.59 ± 0.37	<0.001	a	1.75 / 0.72<br>1.71 ± 0.52	<0.01	a
C20:3*n*-6	24	1.64 / 0.26<br>1.69 ± 0.30		a	1.66 / 0.49<br>1.69 ± 0.39		a	1.79 / 0.51<br>1.85 ± 0.37		a	1.79 / 0.68<br>1.91 ± 0.55		a
	%	15.55 / 8.74		a	18.78 / 19.07		a	12.84 / 20.75		a	18.86 / 27.53		a
	12	14.93 / 2.94<br>14.80 ± 1.95	n.s	a	13.40 / 1.22	0.01	b	13.29 / 2.13	n.s	b	13.72 / 2.37<br>13.57 ± 1.79	n.s	a,b
C20:4*n*-6	24	14.90 / 2.11<br>14.81 ± 1.33		a	14.42 / 1.65		a,b	13.83 / 1.71		b	13.33 / 2.32<br>13.47 ± 1.55		b
	%	0.73 / 9.02		a	4.34 / 9.83		a	2.87 / 10.73		a	0.03 / 11.30		a
	12	2.99 / 1.09<br>2.75 ± 0.63	<0.001	a	2.65 / 0.63	<0.001	a	2.82 / 0.91<br>2.94 ± 0.71	<0.001	a	2.98 / 0.93<br>3.07 ± 0.88	<0.001	a
C22:4*n*-6	24	3.35 / 0.77<br>3.30 ± 0.56		a	3.27 / 1.02<br>3.32 ± 0.61		a	3.59 ± 0.67		a*	3.53. ± 0.73		a
	%	23.32 / 24.77		a	27.15 / 16.47		a	21.56 / 9.05		a	21.13 ± 35.86		a
	12	0.36 / 0.16<br>0.36 ± 0.10	<0.001	a	0.31 / 0.19	<0.001	a,b	0.34 / 0.18	<0.001	a	0.28 / 0.14<br>0.29 ± 0.11	<0.001	b
C22:5*n*-6	24	0.52 / 0.18<br>0.51 ± 0.12		a	0.43 / 0.20		a	0.49 / 0.20		a	0.36 / 0.18<br>0.38 ± 0.13		b
	%	38.39 / 35.95		a	47.08 / 32.38		a	38.37 / 37.19		a	35.82 / 28.92		a

	12	31.58 / 3.83	n.s	a,b	30.22 / 2.21	<0.001	a	31.81 / 2.87<br>31.92 ± 2.16	<0.01	b	33.17 / 3.84<br>33.57 ± 2.55	n.s	c
∑ *n*-6	24	31.74 ± 1.82		a	31.62 / 2.76		a	32.90 / 2.13<br>32.80 ± 1.78		b	33.93 / 3.33<br>33.50 ± 2.65		b
	%	0.77 / 8.04		a	4.36 / 6.78		a	2.66 / 7.39		a	0.73 / 9.32		a
***n*-3 PUFA**													
	12	0.26 / 0.13	<0.001	a	0.24 / 0.23	<0.001	a	0.28 / 0.21	<0.001	a	0.29 / 0.21	<0.001	a
C18:3*n*-3	24	0.17 / 0.09<br>0.18 ± 0.09		a	0.19 / 0.12<br>0.23 ± 0.10		a,b	0.21 / 0.11<br>0.22 ± 0.10		b	0.20 / 0.22<br>0.27 ± 0.19		b
	%	-24.69 ± 33.11		a	-15.64 / 30.03		a	-19.30 ± 42.17		a	-23.36 ± 36.23		a
	12	0.08 / 0.05<br>0.08 ± 0.04	0.03	a	0.07 / 0.04<br>0.08 ± 0.05	0.02	a	0.07 / 0.05	0.02	a	0.06 / 0.03<br>0.06 ± 0.03	n.s	a
C20:4*n*-3	24	0.05 / 0.04<br>0.06 ± 0.03		a	0.06 / 0.05<br>0.07 ± 0.04		a	0.06 / 0.03		a	0.04 / 0.04<br>0.05 ± 0.03		a
	%	-31.05 / 36.31		a	-6.98 / 53.67		a	-27.47 / 36.10		a	-24.70 / 74.49		a
	12	1.08 / 0.56	<0.001	a	0.88 / 0.58	n.s	a,b	0.72 / 0.50	0.04	b.c	0.59 / 0.58	n.s	c
C20:5*n*-3	24	0.74 / 0.52		a	0.76 / 0.56		a	0.63 / 0.47		a,b	0.51 / 0.56		b
	%	-15.35 / 32.80		a	-4.54 / 34.37		a	-7.54 / 26.17		a	-12.59 / 53.63		a
	12	2.73 / 0.69	n.s	a,b	2.49 / 0.64	<0.001	a	2.90 / 0.87<br>2.94 ± 0.60	n.s	b	2.59 / 1.36	n.s	a,b
C22:5*n*-3	24	2.75 ± 0.41		a	2.76 / 0.74		a	3.00 / 0.97<br>2.91 ± 0.54		a	2.60 / 0.82		a
	%	-2.26 / 27.79		a	10.14 / 24.14		b	0.27 / 15.77		a	3.08 / 33.47		a
	12	4.27 / 1.33<br>4.50 ± 0.93	n.s	a	3.98 / 1.37<br>4.01 ± 1.06	<0.01	a	3.13 / 1.34	<0.001	b	2.45 / 1.32	<0.001	b
C22:6*n*-3	24	4.56 / 1.26<br>4.64 ± 0.92		a	4.41 / 1.62<br>4.51 ± 1.12		a	3.60 / 1.22		b	2.95 / 2.80		b
	%	-1.00 / 36.34		a	14.33 / 24.77		a,b	14.06 / 28.48		a,b	20.46 / 53.42		b
	12	5.49 / 1.71	n.s	a	4.80 / 1.52	0.04	a	4.07 / 1.35	0.01	b	3.01 / 1.30	0.01	c
n-3 index	24	5.26 / 1.95		a	5.13 / 1.52		a	4.13 / 1.47		b	3.28 / 2.73		b
	%	-4.85 / 33.28		a	9.68 / 20.62		a,b	8.82 / 21.77		a,b	19.21 / 50.79		b
	12	8.19 / 2.30<br>8.34 ± 1.58	n.s	a	7.12 / 1.74	0.01	b	6.92 / 2.02<br>7.04 ± 1.33	0.04	b	6.27 / 2.03	n.s	c
n-3 LCPUFA	24	8.07 / 1.99<br>8.26 ± 1.21		a	7.89 ± 1.79		a	7.13 / 1.78<br>7.35 ± 1.33		b	5.70 / 3.10		b
	%	-4.13 / 30.86		a	10.18 / 21.46		a	4.47 / 19.05		a	12.63 / 35.15		a
	12	8.60 / 2.24<br>8.69 ± 1.63	n.s	a	7.50 / 1.96	0.01	b	7.25 / 2.05<br>7.44 ± 1.42	n.s	b	6.82 / 2.30	n.s	c
∑ *n*-3	24	8.24 / 2.07<br>8.50 ± 1.27		a	8.17 / 2.05		a	7.43 / 1.95<br>7.63 ± 1.35		b	6.06 / 3.08		b
	%	-6.85 / 27.77		a	8.81 / 21.80		a	3.60 / 19.35		a	11.13 / 36.31		a

	12	40.74 / 4.38	n.s	a	37.99 / 2.53<br>38.42 ± 2.20	<0.001	a	39.08 / 3.55<br>39.36 ± 2.51	<0.01	b	39.25 / 3.95	n.s	b
Σ PUFA	24	40.07 / 2.09		a	40.19 / 1.27<br>40.17 ± 1.25		a	40.47 / 1.92<br>40.43 ± 1.55		a	40.51 / 1.73		a
	%	-0.42 / 11.28		a	6.56 / 6.62		b	3.23 / 7.42		a,b	2.51 / 10.35		a,b
	12	69.37 / 4.87<br>69.77 ± 3.68	n.s	a	71.34 / 4.73<br>70.60 ± 4.87	n.s	a,b	72.69 / 6.39	n.s	b	74.89 / 6.41	n.s	c
%*n*-6[1]	24	71.87/ 6.08<br>70.94 ± 4.00		a	71.68 / 6.50<br>70.56 ± 5.35		a	74.15 / 5.53		a,b	76.23 / 9.12		b
	%	1.75 ± 4.29		a	0.05 ± 5.68		a	0.69 ± 3.11		a*	-1.02 ± 8.13		a
	12	30.63 / 4.87<br>30.23 ± 3.68	n.s	a	28.66 / 4.73<br>29.40 ± 4.87	n.s	a,b	27.31 / 6.39	n.s	b	25.11 / 6.41	n.s	c
%*n*-3[2]	24	28.13 / 6.08<br>29.06 ± 4.00		a	28.32 / 6.50<br>29.44 ± 5.35		a	25.85 / 3.06		a,b	23.77 / 9.12		b
	%	-3.56 ± 9.98		a	0.53± 12.26		a	-1.44 ± 7.98		a	2.10 ± 24.60		a
**TFA**													
	12	0.34 / 0.09	0.001*	a	0.31 / 0.07	0.001	a	0.34 ± 0.09	0.001*	a	0.19 ± 0.04	0.001*	b
Σ TFA	24	0.92 / 0.45<br>0.94 ± 0.34		a	0.88 / 0.69		a	0.84 / 0.52<br>0.88 ± 0.35		a	0.99 / 0.83<br>0.92 ± 0.54		a
	%	182.25 / 133.13		a	170.68 / 303.82		a	181.07 / 140.50		a	296.83 / 415.18		b
	12	0.09 / 0.03<br>0.09 ± 0.02	0.001	a	0.09 /0.03<br>0.09 ± 0.02	0.001	a	0.09 / 0.03<br>0.10 ± 0.03	0.001	a	0.02 / 0.02<br>0.02 ± 0.01	0.001	b
**CLA**	24	0.03 / 0.09		a	0.00 / 0.00		b	0.00 / 0.04		b	0.00 / 0.00		b
	%	-45.00 / 102.78		a	-100.00 / 0.00		b	-100.00 / 53.03		b	-100.00 / 0.00		b
**Ratio**													
	12	3.68 ± 0.65	n.s	a	3.99 / 0.88<br>4.05 ± 0.86	n.s	a,b	4.45 ± 0.91	n.s	b.c	5.45 ± 1.40	n.s	c
n-6/*n*-3	24	3.82 / 1.30<br>3.81 ± 0.71		a	3.92 / 1.03		a	4.65 / 1.34<br>4.44 ± 0.85		b	5.54 / 2.54<br>5.27 ± 1.72		b
	%	3.85 / 23.33		a	-3.99 ± 19.27		a	-0.01 / 17.37		a	-7.38 / 31.78		a
	12	14.08 / 7.91<br>15.66 ± 5.69	0.001*	a	15.62 / 10.83	0.01	b	18.45 / 11.49<br>19.71 ± 7.59	0.01	a,b	24.61 / 22.45	n.s	b
ARA/EPA	24	18.27 / 11.08<br>19.02 ± 6.91		a	18.41 / 12.90		a	22.21 / 12.67<br>21.98 ± 8.15		a,b	25.61 / 31.57		b
	%	19.28 / 39.96		a	15.04 / 43.67		a	10.74 / 33.26		a	14.82 / 61.81		a
	12	3.39 ± 0.67	n.s	a	3.58 / 1.25<br>3.64 ± 1.03	0.01	a	4.47 ± 1.22	<0.001	b	5.68 ± 1.90	<0.001	c
ARA/DHA	24	3.18 / 1.15<br>3.32 ± 0.76		a	3.16 / 1.09		a	3.88 / 1.51<br>4.02 ± 1.14		b	4.64 / 3.25<br>4.74 ± 1.99		b
	%	-4.86 / 27.98		a	-9.37 / 15.20		a,b	-11.40 / 15.94		a,b	-16.63<br>29.31		b
	12	46.34 / 29.75	<0.001	a	47.79 / 36.87	0.01	a	46.67 / 35.91<br>51.60 ± 22.68	n.s	a	45.34 / 25.73<br>47.07 ± 16.99	<0.01	a
LA/ALA	24	66.40 / 28.59		a	56.81 / 40.25		a	57.16 / 17.82<br>58.97 ± 16.70		a	62.58 / 47.87		a

	%	31.61 / 73.82		a	23.65 / 66.74		a	12.63 / 65.32		a	29.17 ± 70.02		a
ARA/LA	12	1.32 / 0.37<br>1.32 / 0.24	n.s	a	1.09 / 0.21<br>1.13 ± 0.18	<0.001	b	1.01 / 0.30	n.s	b.c	0.97 / 0.26<br>0.96 ± 0.20	n.s	c
	24	1.32 / 0.39<br>1.37 ± 0.26		a	1.23 ± 0.18		b	1.09 / 0.19<br>1.11 ± 0.17		c	1.00 ± 0.20		d
	%	2.25 / 14.49		a	9.34 / 20.90		a	5.57 / 19.37		a	4.97 / 25.77		a

Variable expressed as mean (±SD) and/or as median /IQR depending on the statistical test that was performed; ◊ comparison of time points within diet groups; Δ comparison between diet groups at individual time points; groups without a common letter are significantly different, $p < 0.05$; Abbreviations: WD = omnivores, Flex = flexitarians, VG = vegetarians, VN = vegans, m = men, w = women, t = time point (month), % = percentual change from start of intervention, SFA = saturated fatty acids, MUFA = monounsaturated fatty acids, PUFA = polyunsaturated fatty acids, *n* = omega, LC = long-chain, HUFA = highly unsaturated fatty acids, TFA = trans fatty acids, CLA = conjugated linoleic acids, ARA = arachidonic acid, EPA = eicosapentaenoic acid, DHA = docosahexaenoic acid, LA = linoleic acid, ALA = α-linolenic acid

1Percentage of *n*-6 PUFA in fatty acids with three or more double bonds

2Percentage of *n*-3 PUFA in fatty acids with three or more double bonds

Table S3: Estimated Desaturase Activity in erythrocyte lipids during the intervention according to diet group

t	**WD**<br>(27 w, 11 m)	◊	Δ	**Flex**<br>(37 w, 8 m)	◊	Δ	**VG**<br>(30 w, 13 m)	◊	Δ	**VN**<br>(28 w, 14 m)	◊	Δ
**Δ9-desaturase** (C18:1*n*-9/C18:0)												
3	1.239 / 0.333	a	a	1.274 / 0.295<br>1.200 ± 0.353	a	a	1.244 / 0.309<br>1.210 ± 0.255	a	a	1.310 / 0.209	a	a
6	1.182 / 0.236<br>1.196 ± 0.157	a	a	1.238 ± 0.211	a	a	1.232 ± 0.231	a	a	1.231 / 0.339<br>1.225 ± 0.186	a	a
9	1.226 / 0.188<br>1.225 ± 0.143	a	a	1.241 ± 0.172	a	a	1.230 ± 0.180	a	a	1.249 / 0.213<br>1.259 ± 0.154	a	a
12	1.133 / 0.133	a	a	1.248 / 0.218<br>1.249 ± 0.161	a	b	1.231 / 0.187<br>1.232 ± 0.158	a	b	1.261 / 0.199	a	b
%	-8.22 / 24.17		a	0.41 / 27.25		a	2.23 / 26.26		a	-1.16 / 21.86		a
**Δ5-desaturase** (C20:4*n*-6/C20:3*n*-6)												
3	9.903 / 2.912<br>9.87 ± 2.04	a	a	9.679 / 3.392<br>9.829 ± 2.447	a	a	8.887 / 2.962<br>8.902 ± 1.929	a	a	8.777 / 3.529<br>8.652 ± 2.547	a	a
6	10.560 / 3.695<br>10.563 ± 2.462	b,c	a	10.436 / 3.650<br>10.408 ± 2.540	a	a	8.541 / 3.360<br>9.188 ± 2.260	a	b	8.809 / 2.601<br>8.621 ± 2.194	a	b
9	11.012 / 3.588	b	a	9.934 / 3.056<br>10.101 ± 2.269	a	a,b	9.011 / 3.119<br>9.075 ± 1.959	a	b,c	8.740 / 2.909<br>8.648 ± 2.200	a	c
12	10.460 / 3.042	a,c	a	9.900 / 2.799<br>9.987 ± 2.325	a	a,b	8.862 / 3.638<br>8.988 ± 2.152	a	b,c	8.445 / 3.135<br>8.533 ± 2.334	a	c
%	4.27 / 19.84		a	2.93 / 25.85		a	2.96 / 24.83		a	-0.19 / 15.17		a
**Δ5-desaturase** (C20:5*n*-3/C20:4*n*-3)												
3	12.023 / 7.644<br>13.256 ± 5.620	a	a	12.093 / 7.139	a	a	9.762 / 4.727	a	b	8.456 / 4.089	a,c	b
6	11.283 / 5.568<br>11.941 ± 5.108	a	a	11.100 / 7.333	a	a	9.322 / 5.036	a	b	8.035 / 3.559	a	b
9	12.975 / 5.300<br>12.609 ± 4.875	a	a	12.288 / 5.380	a	a	10.000 / 4.587	a	b	10.139 / 6.282	b	a,b
12	11.983 / 4.623<br>11.817 ± 4.301	a	a	13.323 / 6.981	a	a	10.870 / 5.721	a	a	9.986 / 6.159	b,c	a
%	1.55 / 50.20		a	5.84 / 61.05		a	14.63 / 43.39		a	5.86 / 35.82		a
**Δ6-desaturase** (C20:3*n*-6/C18:2*n*-6)												
3	0.112 / 0.030	a	a	0.112 / 0.034	a	a	0.117 / 0.050	a	a	0.103 / 0.026	a	a
6	0.115 / 0.037	a	a	0.108 / 0.041	a	a	0.110 / 0.034	a	a	0.113 / 0.029	a	a
9	0.111 / 0.034	a	a	0.115 / 0.041	a	a	0.116 / 0.038	a	a	0.102 / 0.035	a	a
12	0.131 /0.043	b	a	0.116 / 0.031	a	b	0.126 / 0.041	a	a,b	0.116 / 0.039	a	b
%	13.89 / 27.02		a	2.60 / 24.61		a	2.83 / 27.30		a	10.71 / 32.11		a
**Δ6-desaturase** (C20:5*n*-3/C18:3*n*-3)												
3	4.170 / 2.328	a	a	3.430 / 2.684	a	b	2.496 / 1.876	a,b	b	1.530 / 0.901	a	c
6	3.460 / 2.404	b	a	2.765 / 1.656	a	a	2.254 / 0.962	a	c	1.517 / 0.885	a,b	c
9	4.100 / 2.423	a,b	a	3.026 / 1.911	a	a	2.645 / 1.335	b	b	1.740 / 1.450	a,b	b
12	4.194 / 2.295	a	a	3.080 / 1.603	a	b	2.453 / 1.524	b	c	1.732 / 1.253	b	d
%	-3.67 / 44.93		a	11.09 / 80.87		a	4.93 / 52.13		a	16.87 / 68.72		a

Variable expressed as mean (± SD) and/or as median / IQR depending on the statistical test that was performed; ◊ comparison of time points within diet groups; Δ comparison between diet groups at individual time points; groups without a common letter are significantly different, $p < 0.05$; Abbreviations: WD = omnivores, Flex = flexitarians, VG = vegetarians, VN = vegans, m = men, w = women, t = time point (month), % = percentual change from start of intervention, *n* = omega

Table S4: Estimated Desaturase Activity in erythrocyte lipids from the end of the intervention (12 months) to follow-up (24 months)

t	**WD**<br>(8 m, 19 f)	◊	Δ	**Flex**<br>(5 m, 30 f)	◊	Δ	**VG**<br>(11 m, 27 f)	◊	Δ	**VN**<br>(10 m, 16 f)	◊	Δ
							**Δ9-desaturase** (C16:1*n*-7/C16:0)					
12	0.010 / 0.003	<0.001	a	0.010 / 0.006	<0.001	a	0.010 / 0.003<br>0.010 ± 0.002	<0.001	a	0.006 / 0.004	<0.001	b
24	0.014 / 0.004		a	0.013 / 0.006		a	0.013 / 0.015<br>0.013 ± 0.004		a	0.008 / 0.005		b
%	24.93 / 28.89		a	25.38 / 42.97		a	27.04 / 26.45		a	16.38 / 36.60		a
							**Δ9-desaturase** (C18:1*n*-9/C18:0)					
12	1.125 / 0.108	n.s	a	1.273 / 0.229<br>1.132 ± 0.116	<0.001	b	1.235 / 0.152<br>1.230 ± 0.160	n.s	b	1.215 / 0.194	0.04	a,b
24	1.073 / 0.209<br>1.090 ± 0.157		a	1.132 ± 0.116		a,b	1.190 ± 0.160		b	1.088 / 0.258<br>1.129 ± 0.153		a,b
%	-4.86 / 12.16		a	-8.94 ± 2.64		a	-2.65 ± 11.65		a	-6.91 ± 17.07		a
							**Δ5-desaturase** (C20:4*n*-6/C20:3*n*-6)					
12	10.561 / 2.325<br>10.384 ± 2.011	<0.001	a	9.895 / 2.620<br>9.969 ± 2.524	<0.001	a	8.875 / 3.618<br>9.078 ± 2.140	<0.001	a	8.445 / 4.030<br>8.635 ± 2.712	<0.001	a
24	8.965 ± 1.485		a	8.848 ± 2.237		a	7.966 / 3.040<br>7.803 ± 1.613		b	7.533 ± 2.064		b
%	-13.94 / 11.08		a	-13.38 / 11.08		a	-13.19 / 12.40		a	-14.44 / 15.29		a
							**Δ5-desaturase** (C20:5*n*-3/C20:4*n*-3)					
12	12.031 / 4.651	<0.001	a	13.323 / 7.826	n.s	a	10.951 / 5.397	n.s	a	8.813 / 6.878	<0.001	a
24	14.483 / 8.063		a	14.555 / 8.625		a	12.010 / 7.337		a	13.705 / 7.674		a
%	25.41 / 61.17		a	4.33 / 46.62		a	8.68 / 52.65		a	20.57 / 95.86		a
							**Δ6-desaturase** (C20:3*n*-6/C18:2*n*-6)					
12	0.129 / 0.029	<0.001	a	0.116 / 0.037	<0.001	a	0.124 / 0.040	<0.001	a	0.122 / 0.044	<0.001	a
24	0.146 / 0.027		a	0.145 / 0.046		a	0.144 / 0.041		a	0.135 / 0.036		a
%	16.23 / 16.65		a	26.90 / 28.40		a	15.66 / 25.91		a	21.99 / 29.39		a
							**Δ6-desaturase** (C18:3*n*-6/C18:2*n*-6)					
12	0.003 / 0.002	n.s	a	0.003 / 0.002	n.s	a	0.003 / 0.001	n.s	a	0.003 / 0.001	n.s	a
24	0.004 / 0.002		a	0.003 / 0.002		a	0.004 / 0.002		a	0.003 / 0.002		a
%	0.61 / 51.58		a	-5.05 / 49.12		a	10.26 / 50.95		a	-11.74 / 44.34		a
							**Δ6-desaturase** (C20:5*n*-3/C18:3*n*-3)					
12	4.315/ 1.989<br>4.413 ± 1.498	n.s	a	3.080 / 1.619<br>3.644 ± 1.991	n.s	b	2.640 / 1.427	n.s	b	1.588 / 1.019	0.01	c
24	5.022 / 2.498<br>5.118 ± 1.852		a	3.629 / 1.705		a	2.782 / 1.812		b	1.925 / 1.534		c
%	16.14 / 54.98		a	15.26 / 54.00		a	4.08 / 44.87		a	12.60 / 64.06		a

Variable expressed as mean (± SD) and/or as median / IQR depending on the statistical test that was performed; ◊ comparison of time points within diet groups; Δ comparison between diet groups at individual time points; groups without a common letter are significantly different, $p < 0.05$; Abbreviations: WD = omnivores, Flex = flexitarians, VG = vegetarians, VN = vegans, m = men, w = women, t = time point, % = percentual change from start of intervention, *n* = omega

5

Table S5: Comparison of the percentual change from start of intervention of selected fatty acids within diet groups according to sex, BMI, age, EPA, LA and ARA status

	N	Western diet	◊	Δ	N	Flexitarian	◊	Δ	N	Vegetarian	◊	Δ	N	Vegan	◊	Δ
								**C20:4*n*-6**								
**Women**	27	8.62 / 15.79	n.s	a	37	4.43 / 16.66	n.s	a,b	30	0.84 / 24.56	n.s	a,b	28	4.85 / 15.17<br>4.30 ± 10.77	n.s	a,b
**Men**	11	18.26 / 17.95		a	8	1.53 / 48.79		a	13	-3.16 / 18.73		a	14	8.79 / 19.37<br>8.93 ± 12.31		a
**BMI**<br>< 22.42 kg/m²	9	7.01 / 11.34<br>9.12 ± 7.20	n.s	a	27	4.43 / 16.92	n.s	a	19	7.19 / 20.88	n.s	a	29	8.29 / 17.11<br>6.82 ± 12.09	n.s	a
**BMI**<br>> 22.42 kg/m²	29	12.51 / 21.07<br>13.18 ± 17.29		a	18	2.77 / 28.41		a,b	24	-2.88 / 19.09		b	13	4.63 / 16.49<br>3.66 ± 9.66		a,b
**Age**<br>< 28	13	8.62 / 17.42	n.s	a	24	4.84 / 28.20	n.s	a	22	5.23 / 23.01	n.s	a	25	5.90 / 15.71<br>7.18 ± 12.18	n.s	a
**Age**<br>> 28	25	13.18 / 18.33		a	21	3.09 / 8.36		a,b	21	-3.40 / 19.19		b	17	4.63 / 18.19<br>3.88 ± 10.11		a,b
**EPA**<br>< 0.58 % FAME	15	13.83 ± 12.27<br>13.18 / 22.51	n.s	a	20	4.66 / 49.71	n.s	a	19	3.27 / 22.00	0.04	a	30	8.42 / 16.44<br>7.68 ± 11.49	n.s	a
**EPA**<br>> 0.58% FAME	23	10.73 / 15.84		a	25	3.50 / 14.81		a,b	24	-3.25 / 21.24		b	12	4.21 / 14.58<br>1.26 ± 10.10		a,b
**LA**<br>< 12.59 % FAME	26	12.02 / 16.93	n.s	a	28	3.28 / 24.21	n.s	a	21	0.45 / 23.07	n.s	a	9	5.20 / 21.55<br>4.96 ± 11.12	n.s	a
**LA**<br>> 12.59 % FAME	12	12.61 / 20.06		a	17	4.43 / 16.21		a	22	1.65 / 27.36		a	33	5.90 / 15.57<br>6.09 ± 11.60		a
**ARA**<br>< 13.29% FAME	14	14.03 / 10.63	n.s	a	24	2.77 / 10.86	n.s	a	20	0.49 / 25.04	n.s	a	26	4.89 / 16.04<br>4.41 ± 10.19	n.s	a
**ARA**<br>> 13.29 % FAME	24	7.23 / 18.98		a	21	4.62 / 28.36		a	23	0.45 / 25.26		a	16	8.57 / 19.32<br>8.17 ± 13.08		a
								**C22:4*n*-6**								
**Women**	27	9.17 / 41.38	n.s	a	37	1.22 / 27.50	n.s	a	30	3.59 / 23.<br>27 8.28 ± 21.21	n.s	a	28	5.20 / 23.93<br>1.38 ± 17.68	n.s	a
**Men**	11	15.49 / 51.05		a	8	19.59 / 67.05		a	13	6.19 / 18.08<br>2.13 ± 12.54		a	14	-0.29 / 33.33<br>-0.97 ± 30.47		a
**BMI**<br>< 22.42 kg/m²	9	11.59 / 35.21	n.s	a	27	0.43 /26.69	n.s	a	19	5.03 / 17.72	n.s	a	29	5.56 / 30.32<br>0.89 ± 25.72	n.s	a
**BMI**<br>> 22.42 kg/m²	29	11.18 / 42.57		a	18	6.16 / 39.44		a	24	5.84 / 14.72		a	13	-1.53 / 24.54<br>-0.05 ± 12.98		a
**Age**	13	10.14 / 37.64	n.s	a	24	-1.42 / 34.70	n.s	a	22	5.61 / 25.05	n.s	a	25	5.32 / 21.93	n.s	a

< 28		11.87 ± 22.47								8.75 ± 20.23				1.76 ± 21.08		
**Age**<br>> 28	25	15.49 / 44.72<br>18.92 ± 37.52		a	21	4.22 / 27.99		a	21	3.57 / 13.97<br>3.99 ± 17.94		a	17	0.57 / 30.45<br>-1.11 ± 24.77		a
**EPA**<br>< 0.58 % FAME	15	10.14 / 39.63	n.s	a	20	-1.26 / 64.58	n.s	a	19	6.19 / 19.09<br>12.28 ± 18.55	n.s	a	30	3.83 / 25.13<br>3.42 ± 19.68	n.s	a
**EPA**<br>> 0.58% FAME	23	11.59 / 43.18		a	25	4.22 / 23.10		a	24	3.12 / 18.48 1.79<br>± 18.55		a	12	0.82 / 36.04<br>-6.46 ± 27.77		a
**LA**<br>< 12.59 % FAME	26	10.66 / 44.67	n.s	a	28	0.83 / 34.51	n.s	a	21	2.67 / 18.22	n.s	a	9	6.01 / 37.47<br>9.07 ± 19.37	n.s	a
**LA**<br>> 12.59 % FAME	12	20.76 / 48.30		a	17	8.11 / 28.11		a	22	8.16 / 18.10		a	33	2.58 / 25.59<br>-1.71 ± 22.87		a
**ARA**<br>< 13.29% FAME	14	13.34 / 30.37	n.s	a	24	2.54 / 30.53	n.s	a	20	3.89 / 24.08<br>4.81 ± 21.57	n.s	a	26	3.83 / 25.11<br>-0.46 ± 20.98	n.s	a
**ARA**<br>> 13.29 % FAME	24	8.42 / 47.93		a	21	4.22 / 32.19		a	23	5.03 / 12.50<br>7.82 ± 16.98		a	16	3.07 / 29.11<br>2.31 ± 25.13		a
								**C22:5*n*-6**								
**Women**	27	8.02 / 30.27	n.s	a	37	-2.24 / 37.60	n.s	a,b	30	4.77 / 36.28	n.s	b	28	-9.93 / 35.50<br>-5.75 ± 26.64	n.s	a,b
**Men**	11	12.83 / 38.03		a	8	1.34 / 60.09		a	13	-4.78 / 33.33		a	14	2.31 / 26.33<br>-2.82 ± 20.56		a
**BMI**<br>< 22.42 kg/m²	9	13.01 / 41.11	n.s	a	27	-3.45 / 37.29	n.s	a	19	1.32 / 32.59	n.s	a	29	-1.83 / 31.40<br>-2.43 ± 24.94	n.s	a
**BMI**<br>> 22.42 kg/m²	29	12.42 / 37.76		a	18	1.00 / 37.75		a	24	-3.02 / 30.78		a	13	-10.41 / 31.03<br>-10.00 ± 23.81		a
**Age**<br>< 28	13	7.41 / 21.91<br>8.59 ± 21.50	n.s	a	24	-2.31 / 52.57	n.s	a	22	2.77 / 26.74	n.s	a	25	-2.01 / 29.64<br>-6.70 ± 25.01	n.s	a
**Age**<br>> 28	25	13.01 / 43.03<br>17.92 ± 41.44		a	21	-2.24 / 34.78		a	21	-5.50 / 47.03		a	17	-3.66 / 36.63<br>-1.94 ± 24.37		a
**EPA**<br>< 0.58 % FAME	15	14.36 / 50.57	n.s	a	20	-3.22 / 58.14	n.s	a	19	1.32 / 30.31	n.s	a	30	-1.99 / 26.77	n.s	a
**EPA**<br>> 0.58% FAME	23	11.67 / 27.97		a	25	-1.17 / 32.09		a	24	-3.02 / 36.92		a	12	-16.76 / 49.03		a
**LA**<br>< 12.59 % FAME	26	7.71 / 43.59	n.s	a	28	-5.07 / 37.71	n.s	a	21	-5.52 / 28.48	n.s	a	9	0.90 / 55.19<br>-0.38 ± 32.55	n.s	a
**LA**<br>> 12.59 % FAME	12	12.97 / 23.95		a	17	-0.30 / 34.88		a	22	5.82 / 46.12		a	33	-9.44 / 28.53<br>-5.97 ± 22.39		a
**ARA**<br>< 13.29% FAME	14	12.88 / 15.35<br>15.28 ± 23.43	n.s	a	24	-2.62 / 36.05	n.s	a,b	20	-7.54 / 50.13	n.s	a,b	26	-12.02 / 35.08	0.02	b

**ARA**<br>> 13.29 % FAME	24	4.28 / 47.50<br>14.41 ± 41.89		a	21	-0.30 / 58.87		a	23	1.36 / 22.01		a	16	6.81 / 22.25		a
**C20:5*n*-3**																
**Women**	27	24.56 / 65.87<br>37.38 ± 50.20	n.s	a	37	27.26 / 67.91	n.s	a	30	40.09 / 86.96<br>42.22 ± 50.91	n.s	a	28	34.86 / 90.78<br>44.73 ± 56.46	n.s	a
**Men**	11	35.81 / 87.32<br>38.81 ± 54.73		a	8	25.70 / 98.54		a	13	6.29 / 65.47<br>23.17 ± 44.99		a	14	47.52 / 79.07<br>60.66 ± 48.33		a
**BMI**<br>< 22.42 kg/m²	9	24.56 / 85.43<br>36.64 ± 53.15	n.s	a	27	26.70 / 105.90	n.s	a	19	46.45 / 87.56<br>44.20 ± 47.07	n.s	a	29	35.66 / 82.71<br>43.68 ± 50.05	n.s	a
**BMI**<br>> 22.42 kg/m²	29	31.55 / 69.33<br>38.15 ± 51.01		a	18	29.25 / 47.56		a	24	20.98 / 77.75<br>30.34 ± 51.42		a	13	53.80 / 77.52<br>64.25 ± 61.14		a
**Age**<br>< 28	13	35.81 / 68.45<br>47.30 ± 53.72	n.s	a	24	37.91 / 82.33	n.s	a	22	44.51 / 66.29<br>39.44 ± 46.63	n.s	a	25	35.66 / 82.19	n.s	a
**Age**<br>> 28	25	24.56 / 78.83<br>32.85 ± 49.61		a	21	19.31 / 82.76		a	21	17.31 / 87.69<br>33.34 ± 53.23		a	17	53.80 / 76.23		a
**EPA**<br>< 0.58 % FAME	14	48.79 / 94.70<br>61.60 ± 48.63	0.017	a	20	96.29 / 144.54	n.s	a	19	62.00 / 58.81<br>60.56 ± 41.68	<0.01	a	30	57.88 / 71.60<br>67.72 ± 50.25	<0.001	a
**EPA**<br>> 0.58% FAME	23	22.80 / 91.90<br>22.27 ± 46.87		a	24	19.31 / 55.38		a	24	6.15 / 52.28<br>17.38 ± 47.41		a	12	5.95 / 38.11<br>5.86 ± 34.07		a
**LA**<br>< 12.59 % FAME	26	30.45 / 70.50<br>38.19 ± 51.59	n.s	a	28	42.70 / 88.41	n.s	a	21	30.83 / 80.44<br>43.05 ± 49.15	n.s	a	9	30.80 / 55.97<br>37.91 ± 53.60	n.s	a
**LA**<br>> 12.59 % FAME	12	29.77 / 78.83<br>36.94 ± 51.29		a	17	18.32 / 52.86		a	22	18.28 / 80.51<br>30.18 ± 50.06		a	33	43.82 / 83.59<br>53.35 ± 54.23		a
**ARA**<br>< 13.29% FAME	14	50.28 / 65.58<br>45.86 ± 44.04	n.s	a	24	29.08 / 76.74	n.s	a	20	44.51 / 102.68<br>47.99 ± 52.13	n.s	a	26	40.69 / 84.96<br>46.90 ± 54.51	n.s	a
**ARA**<br>> 13.29 % FAME	24	22.13 / 67.73<br>33.09 ± 54.70		a	21	27.26 / 83.55		a	23	27.38 / 62.25<br>26.44 ± 45.79		a	16	39.74 / 88.37<br>55.16 ± 54.06		a
**C22:5*n*-3**																
**Women**	27	33.43 ± 38.65<br>36.50 / 65.73	n.s	a	37	24.30 / 37.41	n.s	a	30	32.74 / 34.99	n.s	a	28	33.77 / 39.87	n.s	a
**Men**	11	41.76 ± 26.08<br>44.96 / 29.99		a	8	31.42 / 68.73		a	13	24.46 / 26.56		a	14	47.47 / 39.94		a
**BMI**<br>< 22.42 kg/m²	9	32.79 ± 21.63<br>36.50 / 31.90	n.s	a	27	27.51 / 35.10	n.s	a	19	32.87 / 39.96<br>38.43 ± 29.13	n.s	a	29	30.80 / 29.86	n.s	a
**BMI**<br>> 22.42 kg/m²	29	36.80 ± 38.88<br>42.96 / 67.36		a	18	24.33 / 47.01		a	24	28.66 / 31.81<br>29.34 ± 24.83		a	13	51.76 / 46.88		a
**Age**<br>< 28	13	48.85 / 57.13<br>40.95 ± 36.82	n.s	a	24	31.34 / 36.19	n.s	a	22	41.04 / 34.01<br>36.98 ± 28.22	n.s	a	25	41.16 / 35.43	n.s	a
**Age**<br>> 28	25	38.49 / 51.06<br>33.19 ± 35.00		a	21	12.51 / 36.81		a	21	29.87 / 28.79<br>29.56 ± 25.51		a	17	40.49 / 49.77		a

**EPA** < 0.58 % FAME	15	52.93 / 54.29<br>47.73 ± 35.80	n.s	a	20	28.55 / 35.04	n.s	a	19	41.81 / 25.23	0.006	a	30	45.59 / 40.08<br>44.07 ± 27.31	0.003	a
**EPA** > 0.58% FAME	23	32.94 / 43.74<br>28.10 ± 33.54		a	25	14.48 / 38.82		a	24	17.74 / 27.09		a	12	23.07 / 50.94<br>11.63 ± 37.72		a
**LA** < 12.59 % FAME	26	41.80 / 59.88<br>38.24 ± 34.46	n.s	a	28	28.27 / 38.17	n.s	a	21	40.30 / 29.90<br>40.82 ± 25.89	n.s	a	9	30.80 / 25.90<br>34.72 ± 18.19	n.s	a
**LA** > 12.59 % FAME	12	35.72 / 42.02<br>30.66 ± 38.18		a	17	21.44 / 44.38		a	22	23.36 / 29.35<br>26.23 ± 26.40		a	33	40.99 / 46.02<br>34.83 ± 36.94		a
**ARA** < 13.29% FAME	14	41.73 / 50.39<br>33.00 ± 39.88	n.s	a	24	21.13 / 39.76	n.s	a	20	38.60 / 38.84<br>37.50 ± 28.20	n.s	a	26	38.87 / 30.14<br>37.61 ± 28.04	n.s	a
**ARA** > 13.29 % FAME	24	38.57 / 52.39<br>37.51 ± 33.19		a	21	27.51 / 39.52		a	23	29.66 / 28.21<br>29.76 ± 25.74		a	16	40.82 / 55.01<br>30.24 ± 41.72		a
**C22:6*n*-3**																
**Women**	27	29.64 / 49.67<br>26.40 ± 29.45	n.s	a	37	19.90 / 37.91	n.s	a	30	17.83 / 37.86<br>21.48 ± 31.30	n.s	a	28	16.21 / 49.53	n.s	a
**Men**	11	24.56 / 53.97<br>24.00 ± 34.43		a	8	7.36 / 65.32		a	13	17.49 / 58.41<br>4.15 ± 32.32		a	14	32.82 / 36.63		a
**BMI** < 22.42 kg/m²	9	31.61 / 60.82<br>27.76 ± 35.90	n.s	a	27	18.39 / 36.57	n.s		19	22.80 / 35.14<br>24.63 ± 30.17	n.s		29	20.89 / 46.48	n.s	a
**BMI** > 22.42 kg/m²	29	24.56 / 49.13<br>25.07 ± 29.33		a	18	9.95 / 40.40			24	14.20 / 41.88<br>9.60 ± 32.89			13	19.04 / 71.46		a
**Age** < 28	13	24.56 / 39.07<br>23.37 ± 24.16	n.s	a	24	20.32 / 38.64	n.s		22	22.43 / 36.90<br>18.55 ± 37.79	n.s		25	18.13 / 45.47	n.s	a
**Age** > 28	25	27.42 / 55.77<br>26.92 ± 33.75		a	21	8.89 / 45.38			21	14.01 / 29.17<br>13.82 ± 25.91			17	26.13 / 52.19		a
**EPA** < 0.58 % FAME	15	32.76 / 56.72<br>37.48 ± 31.77	0.047	a	20	35.39 / 142.61	<0.01	a	19	22.80 / 48.78	0.02	a	30	25.79 / 36.77	n.s	a
**EPA** > 0.58% FAME	23	10.41 / 50.34<br>18.03 ± 27.70		a	25	8.47 / 32.30		a	24	5.35 / 43.73		a	12	5.91 / 69.33		a
**LA** < 12.59 % FAME	26	17.41 / 48.16<br>23.29 ± 28.12	n.s	a	28	10.25 / 31.52	n.s	a	21	14.76 ± 33.14<br>14.01 / 35.28	n.s	a	9	10.86 / 41.32	n.s	a
**LA** > 12.59 % FAME	12	35.99 / 67.71<br>30.94 ± 35.92		a	17	30.73 / 78.99		a	22	21.82 / 33.36<br>17.66 ± 32.08		a	33	26.13 / 46.75		a
**ARA** < 13.29% FAME	14	47.95 / 58.73<br>37.87 ± 30.95	n.s	a	24	23.44 / 38.30	n.s	a	20	22.59 / 36.57<br>21.13 ± 29.38	n.s	a	26	18.58 / 48.35	n.s	a
**ARA** > 13.29 % FAME	24	11.73 / 36.89<br>18.61 ± 28.53		a	21	8.94 / 36.37		a	23	14.01 / 30.46<br>11.99 ± 34.62		a	16	29.46 / 48.16		a
***n*-3 LCPUFA**																
**Women**	27	35.34 / 37.84<br>28.09 ± 28.41	n.s	a	37	24.85 / 33.24	n.s	a	30	25.73 / 33.38<br>27.25 ± 28.69	n.s	a	28	16.45 / 45.64	n.s	a

**Men**	11	34.06 / 63.49<br>31.21 ± 30.38		a	8	15.04 / 63.22		a	13	16.43 / 32.47<br>14.39 ± 23.54		a	14	33.13 / 34.68		a
**BMI**<br>< 22.42 kg/m²	9	32.51 / 51.91<br>29.06 ± 29.80	n.s	a	27	26.89 / 34.95	n.s	a	19	27.59 / 30.08<br>30.49 ± 26.20	n.s	a	29	24.79 / 30.50<br>27.65 ± 26.76	n.s	a
**BMI**<br>> 22.42 kg/m²	29	35.34 / 43.73<br>28.97 ± 28.78		a	18	19.76 / 34.09		a	24	17.27 / 37.85<br>17.72 ± 27.92		a	13	40.34 / 59.56<br>43.04 ± 50.23		a
**Age**<br>< 28	13	40.96 / 45.24<br>31.12 ± 28.89	n.s	a	24	28.40 / 30.40	n.s	a	22	28.11 / 35.84<br>26.36 ± 31.59	n.s	a	25	22.49 / 45.79	n.s	a
**Age**<br>> 28	25	34.06 / 46.83<br>27.88 ± 29.00		a	21	14.44 / 38.50		a	21	16.90 / 31.19<br>20.22 ± 23.08		a	17	34.18 / 39.37		a
**EPA**<br>< 0.58 % FAME	15	45.26 / 43.79<br>41.74 ± 26.03	0.02	a	20	39.67 / 65.03	<0.001	a	19	34.26 / 30.79	<0.01	a	30	37.29 / 44.36	<0.01	a
**EPA**<br>> 0.58% FAME	23	15.56 / 51.91<br>20.68 ± 27.62		a	25	14.01 / 33.70		a	24	10.68 / 31.50		a	12	12.58 / 26.18		a
**LA**<br>< 12.59 % FAME	26	33.92 / 47.35<br>29.24 ± 28.59	n.s	a	28	25.87 / 26.51	n.s	a	21	21.09 / 32.04<br>25.86 ± 25.75	n.s	a	9	15.88 / 38.50<br>21.49 ± 23.18	n.s	a
**LA**<br>> 12.59 % FAME	12	37.51 / 44.71<br>28.47 ± 29.93		a	17	16.54 / 53.35		a	22	19.18 / 34.89<br>20.98 ± 29.68		a	33	31.92 / 44.57<br>35.39 ± 38.24		a
**ARA**<br>< 13.29% FAME	14	43.39 / 26.16<br>36.25 ± 29.57	n.s	a	24	22.56 / 38.01	n.s	a	20	29.84 / 33.94<br>29.21 ± 26.92	n.s	a	26	24.50 / 29.71<br>29.97 ± 29.45	n.s	a
**ARA**<br>> 13.29 % FAME	24	16.40 / 47.49<br>24.76 ± 27.79		a	21	22.35 / 32.36		a	23	16.90 / 31.07<br>18.27 ± 27.77		a	16	37.26 / 50.73<br>36.38 ± 44.99		a

Variable expressed as mean (±SD) and/or as median /IQR depending on the statistical test that was performed; ◊ comparison of time points within diet groups; Δ comparison between diet groups at individual time points; groups without a common letter are significantly different, $p < 0.05$; Abbreviations: N = sample size, BMI = Body mass index, PUFA = polyunsaturated fatty acids, LC = long-chain, EPA = eicosapentaenoic acid, LA = linoleic acid, ARA = arachidonic acid, *n* = omega

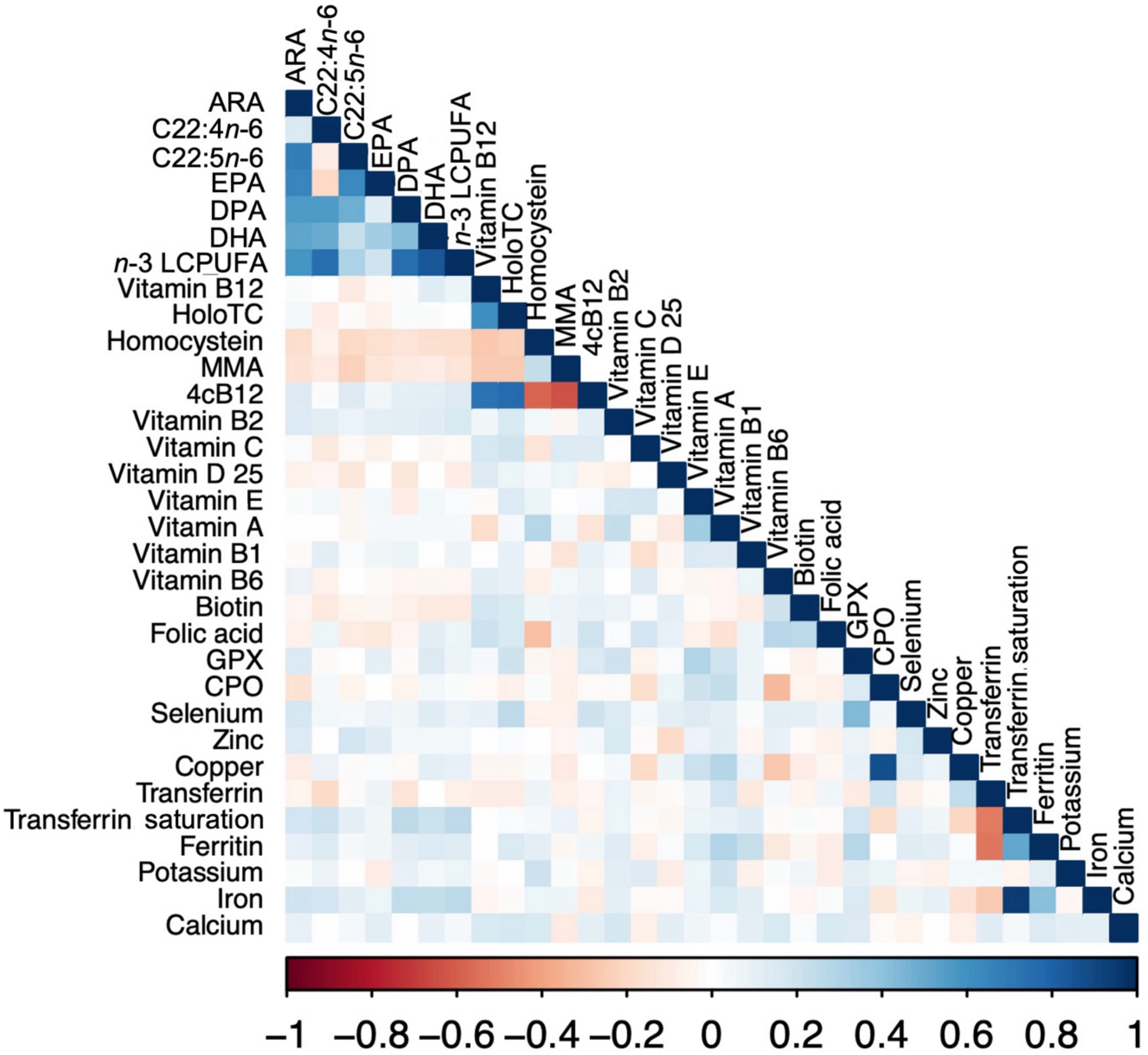

Figure S1: Correlation matrix of the percentual change of selected fatty acids in erythrocyte lipids and biomarkers of micronutrients at the beginning of the intervention according to Spearman´s rank correlation. ARA = arachidonic acid, EPA = eicosapentaenoic acid, DPA = docosapentaenoic acid, DHA = docosahexaenoic acid, PUFA = polyunsaturated fatty acids, *n* = omega, HoloTC = Holo-transcobalamin, MMA = methyl malonic acid, 4cB12 = score of combined index for Vitamin B_{12} deficiency, GPX = glutathione peroxidase, CPO = ceruloplasmin oxidase
